# Identification of mycobacterial Thymidylate kinase inhibitors: a comprehensive pharmacophore, machine learning, molecular docking, and molecular dynamics simulation studies

**DOI:** 10.1007/s11030-024-10967-w

**Published:** 2024-08-16

**Authors:** Rupesh V. Chikhale, Surbhi Pravin Pawar, Mahima Sudhir Kolpe, Omkar Dilip Shinde, Kholood A. Dahlous, Saikh Mohammad, Pritee Chunarkar Patil, Shovonlal Bhowmick

**Affiliations:** 1 Department of Pharmaceutical and Biological Chemistry, School of Pharmacy, University College London, London, UK; 2 SilicoScientia Private Limited, Nagananda Commercial Complex, No. 07/3, 15/1, 18th Main Road, Jayanagar 9th Block, Bengaluru, 560041 India; 3 Department of Bioinformatics, Rajiv Gandhi Institute of IT and Biotechnology, Bharati Vidyapeeth (Deemed to be University), Pune-Satara Road, Pune, India; 4 Department of Chemistry, College of Science, King Saud University, 11451 Riyadh, Saudi Arabia

**Keywords:** Mycobacterium tuberculosis, Thymidylate kinase, Machine learning, Pharmacophore, Virtual screening, Molecular docking, Molecular dynamics

## Abstract

Thymidylate kinase (TMK) is a pivotal enzyme in Mycobacterium tuberculosis (Mtb), crucial for phosphorylating thymidine monophosphate (dTMP) to thymidine diphosphate (dTDP), thereby playing a critical role in DNA biosynthesis. Dysregulation or inhibition of TMK activity disrupts DNA replication and cell division, making it an attractive target for anti-tuberculosis drug development. In this study, the statistically validated pharmacophore mode was developed from a set of known TMK inhibitors. Further, the robust pharmacophore was considered for screening the Enamine database. The chemical space was reduced through multiple molecular docking approaches, pharmacokinetics, and absolute binding energy estimation. Two different molecular docking algorithms favor the strong binding affinity of the proposed molecules towards TMK. Machine learning-based absolute binding energy also showed the potentiality of the proposed molecules. The binding interactions analysis exposed the strong binding affinity between the proposed molecules and active site amino residues of TMK. Several statistical parameters from all atoms MD simulation explained the stability between proposed molecules and TMK in the dynamic states. The MM-GBSA approach also found a strong binding affinity for each proposed molecule. Therefore, the proposed molecules might be crucial TMK inhibitors for managing Mtb inhibition subjected to in vitro/in vivo validations.

**Supplementary Information:**

The online version contains supplementary material available at 10.1007/s11030-024-10967-w.

## Introduction

Tuberculosis (TB) is a transmissible and airborne infectious disease that mainly occurs in the lungs and can also spread to other body parts [1]. It is well-established that *Mycobacterium tuberculosis* (Mtb) is the etiologic agent for TB [2]. TB has already been known as the top cause of death worldwide, which killed 1.3 million people in 2023. It was reported by the World Health Organization (WHO) in 2022 that almost two billion people, which is one-quarter of the total population across the globe, were latently infected by Mtb. There is about a 5—10% possibility for reactivation of TB in the latent TB-infected individuals [3]. On the other hand, the dormant Mtb may be activated in immunocompromised patients, such as individuals co-infected with the human immunodeficiency virus (HIV) [4, 5]. About 18 times higher is the chance of getting Mtb infection by individuals living with HIV than by persons without HIV [6]. A great matter of concern is that around 1.3 million patients succumbed each year due to TB [7]. In addition to the above concerns, drug resistance is a significant obstacle to controlling or treating TB. In multidrug-resistant TB (MDR-TB) TB, both isoniazid and rifampicin are resistant [8]. It is already evident that MTB-TB has a crucial contribution to Post-TB Lung Disease (PTLD), which leads to disability and requires rehabilitation [9]. Moreover, extensively drug-resistant TB (XDR-TB) is a highly challenging infection that develops resistance to rifampicin and any fluoroquinolone along with a priority drug A such as bedaquiline or linezolid [10–12]. Although about 30 TB drugs are used for the TB treatment, still such as armamentarium is not adequate to handle the MDR-TB and XDR-TB [13].

Thymidylate kinase (TMK) of Mtb is crucial for DNA synthesis, and it can be an important protein target for identifying potential anti-TB molecules [14]. TMK is an essential protein that catalyses the phosphorylation of thymidine monophosphate (dTMP) to thymidine diphosphate (dTDP) using adenosine triphosphate (ATP) as the source of the phosphoryl group [15, 16]. The essential thymidine triphosphate (dTTP) level is maintained through TMK [17]. Further, dTTP is known to be the building block of DNA, which is required for DNA replication. Hence, inhibition of MTK is one of the critical strategies for stopping the replication that leads to killing the Mtb cells. Moreover, the sequence of TMK from Mtb is about only 22% of the identity of the sequence of human TMK [18]. The above low sequence identity explained that selective inhibitors of Mtb TMK might be safe and harmless for human cells.

Advancements in computational power and computational chemistry tools are widely involved in identifying the potential molecules targeting the various Mtb proteins [19–22]. Moreover, several *in-silico* studies on Mtb TMK based on pharmacophore [23], virtual screening [24], and molecular docking [25] have been reported. Hence, the present study was considered to develop the pharmacophore space modelling and screening of the Enamine kinase database (https://enamine.net/compound-libraries/targeted-libraries/kinase-library) through a validated pharmacophore model. Several crucial approaches in terms of multiple molecular docking, pharmacokinetics and toxicity, and machine learning-based absolute binding energy were used to narrow down the chemical space. Further, the dynamic stability of the final molecules bound with MTK was assessed through all atoms molecular dynamics (MD) simulation.

The motivation behind this study is rooted in the urgent need for new TB treatments, especially those effective against drug-resistant strains. By targeting TMK, this research aims to contribute to developing novel therapies that can enhance treatment outcomes and reduce the global burden of TB. Ultimately, this study underscores the potential of computational methods to accelerate drug discovery efforts against TB, offering hope for improved therapies in the fight against this persistent global health threat.

## Materials and methods

### Dataset collection and preparation for Thymidylate kinase inhibitors

A dataset of 94 compounds analogue of Mtb TMK was retrieved from the BindingdB database (https://www.bindingdb.org/rwd/bind/index.jsp) along with their inhibitory concentration (IC_50_) value and structures in structure data file (sdf) format. The IC_50_ value of downloaded compounds was in the range of 1 to 1230000 nm. The IC_50_ was converted into the logarithm value using the equation

1 $$ {\text{pIC}}50 \, = \, - \log \left( {{\text{IC}}50 \, \times \, 10^{ - 9} } \right) $$

To divide the dataset into training and test sets, the Li and Hoffman approach was considered which can be explained as (i) the selected molecules should be rich in structural features and activity range, (ii) to overcome the chance correlation and ensure the statistical significance, each training set must contain at least 16 molecules, (iii) most and least active compounds of the dataset must be part of each training set, and (iv) at least four orders of magnitude in molecule’s biological activity should present [26]. Following the above criteria, four training and test sets were generated, with 30 and 64 molecules in each set, respectively, given in Table S1 (Supplementary file). The coordinates and energy minimization of each compound in the dataset were corrected using the modified CHARMM force field [27].

### 3D-QSAR pharmacophore model generation

A pharmacophore space model was generated using the ‘3D QSAR Pharmacophore Model Generation module in the Discovery Studio (DS) program [28]. Each training set molecule had conformations generated by the DS software package’s ‘Cat-Conf’ function. The BEST strategy was chosen from among the available options to obtain several appropriate conformations by extracting energy minimization and optimization utilizing the poling algorithm, ensuring thorough and enhanced coverage of the conformational. In its application, the BEST algorithm involves the spatial arrangement of chemical features instead of the mere arrangement of atoms. ‘Feature Mapping’ predicted the favourable features for highly active compounds within the dataset. The identified features from the mapping process were subsequently utilised as input features for generating the pharmacophore model. Various parameters, including spacing and uncertainty, were fine-tuned to formulate the optimal hypothesis. The spacing parameter denotes the minimum permissible distance between inter-features within the hypothesis. Concurrently, weight variation signifies the range of magnitudes explored by the hypothesis, wherein each feature contributes to indicating the compound’s biological activity. The default value for spacing is 3.0. However, this value can vary between 3.0 and 1.0, contingent upon specific cases. The uncertainty parameter reflects the estimation error, representing the error cost’s standard deviation. The default value for the uncertainty parameter is 3, but it may fluctuate between 1.5 and 4.0 depending on the context.

The 3D QSAR pharmacophore generation protocol employs the ‘HypoGen’ algorithm to formulate Structure–Activity Relationship (SAR) hypothesis models (pharmacophores) based on a set of ligands with known activity values against a specific biological target. The input ligands must possess two molecular properties: known activity and uncertainty. The uncertainty factor assigned to each compound reflects the extent of uncertainty in the activity value, considering the expected statistical distribution of biological data collection. A total of five pharmacophoric features, namely hydrogen bond (HB) acceptor (‘a’), HB donor (‘d’), hydrophobic (‘p’), ring aromatic (‘r’), and positive ionizable (‘pi’) were selected to generate a maximum of ten pharmacophores, while the remaining parameters were maintained at their default values. The activity of each compound in the training set is estimated through regression parameters derived from regression analysis, utilizing the relationship between geometric fit values and the negative logarithm of activity. A higher geometric fit value corresponds to a greater predicted activity for the compound. Pharmacophore models were developed using all four sets, and subsequent statistical analyses were conducted based on both training and test set molecules.

### Validation of pharmacophore model

Validation of any in-silico model is crucial to ascertain its predictive capability, applicability, and overall robustness. In this study, the chosen pharmacophore models derived from the training sets underwent validation through several distinct approaches, such as internal validation, test set prediction, cost function analysis, Fischer’s randomization test, and decoy set validations. These comprehensive validation procedures aim to ensure the reliability and effectiveness of the developed pharmacophore models in predicting molecular interactions and activities.

The pIC_50_ of the training set molecules was predicted through the selected model and the Leave-One-Out (LOO) cross-validation coefficient was calculated as per Eq. (2) [29]. The root-mean-square error (rmse) was calculated using Eq. (3).

2 $$ Q^{2} = 1 - \frac{{\sum \left( {Y_{{{\text{pred}}}} - Y} \right)^{2} }}{{\sum \left( {Y - \overline{Y}} \right)}} $$

3 $$ {\text{rmse}} = \sqrt {\frac{{\sum \left( {Y_{{{\text{pred}}}} - Y} \right)^{2} }}{n}} $$

*Y*, *Y*_pred_ and $\overline{Y }$ are the observed, predicted, and mean pIC50 of the training set. n is the number of compounds in the training set. A high Q2 value and low rmse indicate the model’s better predictive ability.

A dedicated test set was meticulously selected to assess the robustness and predictive performance of the developed pharmacophore model, ensuring diversity in chemical structures and bioactivities. This independent test set served as a critical benchmark to evaluate the model’s ability to generalize beyond the compounds used for its development. The pharmacophore model was applied to the compounds within the test set, predicting their biological activities based on identified pharmacophoric features. The accuracy of these predictions was rigorously evaluated using well-established performance metrics such as *R*^2^_pred_ and rmse (Eq. 3) [29]. The mathematical expression of R^2^_pred_ is given in Eq. (4).

4 $$ R_{{{\text{pred}}}}^{2} = 1 - \frac{{\sum \left( {Y_{{{\text{pred}}\left( {{\text{test}}} \right)}} - Y_{{\left( {{\text{test}}} \right)}} } \right)}}{{\sum \left( {Y_{{\left( {{\text{test}}} \right)}} - \overline{Y}_{{{\text{training}}}} } \right)}} $$

In the above expression, *Y*_pred(test)_ and *Y*_(test)_ represent the predicted and observed pIC50 of the test set compounds. *Y*_training_ is recognized as the mean activity of the training set compounds. It is reported that the *R*^2^_pred_ of any predictive model with more than 0.50 can be considered as acceptable robustness of the molecules. The rmse of the test can be calculated by Eq. (3) by replacing *Y*_pred_, *Y* and *n* parameters for test set compounds.

Cost-function analysis plays a pivotal role in developing and selecting a robust pharmacophore model. Cost-function analysis comprises three terms: weight cost, error cost, and configuration cost. The fluctuation of the weight variation with the input actual value signifies the weight cost. The error cost can explain the difference between the predicted and observed activity of the training set. The complexity of the hypothesis space determines a fixed cost. Configuration cost, indicating the entropy of the hypothesis space, is deemed satisfactory when its value is below 17 for a robust pharmacophore model. The null cost (Δ) is the difference between the cost of the null hypothesis (null cost) and the total cost, and any pharmacophore model having Δ cost more than 60 signifies that the hypothesis does not merely reflect a chance correlation.

In validating pharmacophore models, the Fischer randomization test [30] was employed as an essential statistical tool. This test assesses the robustness and significance of the developed pharmacophore model by evaluating its performance against randomly generated datasets. The primary aim is to ascertain whether the observed correlation in the original model is statistically significant and not merely a chance occurrence. During the Fischer randomization test, the biological activity values associated with the compounds in the dataset are randomly shuffled, disrupting any inherent structure or correlation between the features and activities. The pharmacophore model is then reapplied to these randomized datasets, generating a distribution of correlation coefficients assuming no true correlation exists between features and activities. The observed correlation coefficient from the original model is then compared to the distribution obtained from the randomization process. If the observed correlation falls within the tails of the randomized distribution, it suggests that the original correlation is likely a result of chance and is not statistically significant. Conversely, if the observed correlation lies outside the distribution’s tails, it indicates that the pharmacophore model has captured a meaningful relationship between molecular features and biological activity.

A rigorous validation was conducted employing the decoy set approach to evaluate the screening efficacy of the chosen pharmacophore model. This method assesses the model’s capability to distinguish between active and inactive molecules. Decoys were generated for the initial 25 active compounds obtained from the original dataset to achieve this. The decoys were systematically created using the DUD-E database generator (https://dude.docking.org/generate), ensuring their physical resemblance to active inhibitors while maintaining chemical distinctions to prevent biases in the enrichment factor calculations. The selection of decoys was guided by five essential parameters, namely molecular weight, number of rotational bonds, hydrogen bond donor count, hydrogen bond acceptor count, and the octanol–water partition coefficient of the active inhibitors. Subsequently, the pIC50 of the active and decoy molecules was calculated. Based on the predicted pIC50, the molecules were categorized as true positive (TP), true negative (TN), false positive (FP), and false negative (FN). The above confusion matrix generated the data receiver operating characteristic (ROC) curve and area under the curve (AUC).

### Virtual screening

Virtual screening is a pivotal technique for identifying novel and potent compounds capable of interacting with specific receptor sites to modulate activity. In this study, compounds were sourced from the kinase library within the Enamine Database, yielding a total of 64,960 compounds. After extracting a distinct database from the original 64,960 compounds, a meticulous validation process ensued. The ‘Search 3D Database” module of the DS was used to screen the Enamine databases, keeping the limit of 3000. The outcome of this screening furnished a collection of sorted compounds. Pharmacokinetic assessment and absolute binding free energy calculation were carried to screen the molecular docking further using Autodock vina1.2 (ADV) [31] and PLANTS (Protein–Ligand ANT System) [32].

### Molecular docking using Autodock vina

Molecular docking involves the computational exploration of a molecular search space defined by the method’s molecular representation. It entails ranking candidate solutions to identify optimal binding modes, necessitating both a search method and a scoring function. Primarily applied in ligand–protein docking, molecular docking has extended its utility to include protein–protein docking. Widely employed in drug discovery, it contributes to various aspects such as structure–activity studies, lead optimization, virtual screening for lead identification, facilitating mutagenesis predictions, aiding in substrate and inhibitor fitting to electron density in x-ray crystallography, chemical mechanism studies, and assisting in combinatorial library design. Molecular docking was performed using ADV to screen out the molecules having high-affinity and low binding energy. The ADV is an open-source, widely used molecular docking engine that uses a Monte Carlo (MC) [33] based search algorithm to explore the equal efficiency for its energy landscape. Validation of the molecular docking protocol is an essential step to verify the best-docked pose as comparable to the crystalized conformation [34]. For this purpose, the co-crystal ligand, TMP, was re-drawn and docked at the active site where the co-crystal ligand was bound. On successful docking, the best-docked pose was superimposed on a co-crystal ligand conformer, and RMSD was recorded [35]. The above step was repeated by varying the parameters. It is reported that the RMSD of ≤ 2 Å between best-docked pose and crystal conformation indicates the possibility of generation comparable conformation similar to the crystalization.

Prior to the docking, the crystal structure of TMK was obtained from the RCSB-Protein Databank (PDB) [36] having the PDB ID: 1G3U [37]. The selected protein structure consists of 214 amino acids with resolution and R-value of 1.95 Å and 0.250, respectively. The Autodock tool (ADT) was used to prepare the protein by repairing the missing atoms, adding hydrogens and Gasteiger charge, and saving in.pdbqt format, then assigning the AD4 atom types.

The small molecules were prepared using the OpenBabel tool. In particular, the molecules were converted into 3D format, added hydrogen and Gasteiger charges, and saved as.pdbqt and.mol2 formats for docking in ADV and PLANTS, respectively.

### ADME and toxicity prediction

The interplay among pharmacokinetics, toxicity, and potency is a pivotal aspect in developing effective drugs. A compound’s pharmacokinetic profile delineates its absorption, distribution, metabolism, and excretion (ADME) properties. This study uses graph-based structural signatures to examine and predict various ADMET properties for novel chemical entities. Our findings demonstrate the successful utilization of these signatures in training predictive models for diverse ADMET properties. Termed pkCSM [38], this approach not only facilitates the development of predictive models but also offers a platform for analyzing and enhancing pharmacokinetic and toxicity properties. The pkCSM tool is accessible through a user-friendly and freely available web interface ( https://biosig.lab.uq.edu.au/pkcsm/), providing medicinal chemists with a valuable resource to strike a balance between potency, safety, and pharmacokinetic attributes. A number of parameters were assessed, including intestinal absorption, skin permeability, BBB, central nervous system (CNS) permeability, AMES toxicity, hepatotoxicity, skin sensitivity, minnow toxicity, and maximum tolerated dosage. Molecules found to show an acceptable ADMET profile were taken for further analyses.

### PLANTS docking and absolute binding energy estimation

The PLANTS binding score was employed as a crucial parameter for evaluating the interactions between protein and ligand molecules. In the experimental procedures, the PLANTS binding score was utilized as a quantitative measure to assess the strength and suitability of the protein–ligand interactions. With its multifaceted components, this score offers a comprehensive evaluation of the binding affinity, considering factors such as molecular conformations, torsional potentials, clashes, and the ligand’s spatial orientation within the binding site. Molecules retained after pharmacokinetics and toxicity assessment were considered for molecular docking using PLANTS and, subsequently, absolute binding affinity analyses through K_Deep_. For molecular docking, PLANTS uses the MIN–MAX ANT System which is based on the Ant Colony Optimisation (ACO) algorithm [32]. This algorithm uses a probabilistic technique for solving computational problems which can be reduced to finding good paths through graphs. The solution gets generated through the empirical scoring function with a high value of the pheromone parameter receiving the best solution. K_Deep_ is a machine-learning approach for absolute binding affinity prediction, utilizing advanced 3D-convolutional neural networks. It is readily accessible, providing users with a straightforward means to assess their protein − ligand complexes [39].

### MD simulation

The final selected molecule, TMP, and most active compounds bound with TMK were considered for 100 ns of MD simulation using Gromacs2023.2. The protein topology was generated using the CHARMM36 force field [40]. SwissParam was used for the ligand topology. The protein–ligand complex system was confined in a cubic box having a distance of 10 Å from the center to the edge of the box. For each system, the time step, constant pressure, and constant temperature of 2 fs, 1 atm, and 300 K, respectively, were considered to conduct the simulation. The required number of Na + or Cl– was added to neutralize each system. To eliminate the close contact and overlapping, all the systems separately were minimized for 50,000 steps using the steepest descent algorithm. The hydrogens were restricted using the SHAKE algorithm, whereas Particle Mesh Ewald (PME) [41] was used to address the long-range electrostatic interactions. The non-bonded interaction cut-off value was set at 8 Å. The overall temperature of the simulation was regulated through the Langevin thermostat with a 2.0 collision frequency. The Monte Carlo barostat at 1 atm has the volume exchanges occurring every 100 fs considered for pressure regulation of the system. Each system was equilibrated to distribute the solvent equally through NVT (number of particles, volume, and temperature) followed by NPT (number of particles, pressure, and temperature) ensembles for 5 ns of each. On successful completion of the simulation, a number of statistical parameters were extracted, such as protein backbone and ligand RMSD, root-mean-square fluctuation (RMSF), radius of gyration (RoG), inter-molecular hydrogen bonds, and free energy landscape (FEL).

The binding affinity of each molecule in terms of binding free energy (Δ*G*_bind_) was explored from the MD simulation trajectories through the Generalised Born and Surface Area Solvation (MM-GBSA) method [42]. The gmx_MMPBSA package used 2000 frames from the entire trajectory with an interval of 5, [43] and the ΔG_bind_ was estimated. Our previous publications have discussed the explanation and expression of the MM-GBSA approach [44, 45].

## Results and discussion

### Training and test set selection

The entire set of 94 TMK inhibitors was considered to generate four training and test sets (Tr1 and Ts1; Tr2 and Ts2; Tr3 and Ts3; and Tr4 and Ts4). Each training set was used to develop the pharmacophore model, and the corresponding test set was used to evaluate the predictivity of the model. The entire set with the molecules categorized in different training and test sets is given in Table S1 (Supplementary file). The pharmacophore model from each training set was developed and validated using the corresponding test set to select the best training set and corresponding test set. Spacing and uncertainty varied in each run number for the model development, and different statistical parameters were recorded. For each run, a total of 10 hypotheses were generated with the statistical parameters. The best hypothesis of each run was extracted according to the R, RMSD, costs, etc., and given in Table S2 (Supplementary file). It can be noted that for each run number, Hypo #1 was found to be the best hypothesis. From Table S2, it can be seen that Tr1 and Ts1 were given the best statistical parameters. Hence, Tr1 and the corresponding test set, i.e., Ts1, were considered the final training and test set, and subsequently, the best model was validated and used to screen the chemical database. Observed and predicted biological activity are given in Table 1.

**Table 1:** Hypothesis parameters observed in successive runs for TMK inhibitors

Run No	Hypo no	Spacing	^a^Unc	^b^Wt. Var	^c^R	Rmsd	Costs					Features	Q^2^_test_
							Total	Null	Fixed	^d^∆	^e^Config		
1	Hypo #1	3.0	3.0	0.3	0.76	2.04	183.00	251	119.00	68.00	16.40	a, d, 3p	5.070
2	Hypo #1	2.5	3.0	0.3	0.85	1.63	161.50	251	119.00	89.50	16.17	a, d, 3p	5.150
3	Hypo #1	2.0	3.0	0.3	0.81	1.69	163.40	251	119.60	131.4	16.60	a, d, 3p	0.622
4	Hypo #1	1.5	3.0	0.3	0.80	1.86	175.00	251	119.60	76.00	16.60	2a, 3p	0.354
5	Hypo #1	1.0	3.0	0.3	0.82	1.81	174.00	251.02	120.00	77.00	16.60	a, d, 3p	0.504
6	Hypo #1	2.0	2.5	0.3	0.82	2.16	363.80	622	209.30	259.00	16.20	a, d, 3p	0.426
7	Hypo #1	2.0	2.0	0.3	0.86	2.52	214.10	464	105.90	250.00	16.70	a, d, 3p	0.342
8	Hypo #1	2.0	1.5	0.3	0.82	4.89	466.30	1173	89.80	706.70	16.70	a, d, 2p	0.450
9	Hypo #1	2.0	3.0	0.3	0.85	1.69	163.40	25	119.60	131.40	16.60	a, d, 3p	0.524

^a^Uncertainty

^b^Weight Variation

^c^Correlation coefficient

^d^(Null Cost-Total cost)

^e^Configuration cost

### Pharmacophore model development

The considered best training set (Tr1) was used to develop the pharmacophore by varying the input parameters. The statistical data of each set of input parameters are given in Table 1. In particular, a total of 9 sets of parameters were used to generate the model, and each parameter set can be identified as Run no. Table 1 explains that the correlation (R) and cost functions were changed with the parameter variation. The *Q*^2^_test_ is the correlation coefficient between the observed and experimental of the test set compounds. Although Hypo #1 of Run no. 7 showed the highest R (Table 1), the Q^2^_test_ was found to be less than 0.50. Several models were developed with a slightly higher R-value, but the *Q*^2^_test_ was found to be either less than the acceptable value (0.50) or just crossed it. Hypo #1 of Run no. 3 with spacing, uncertainty, and weight variation of 2.0, 3.0, and 0.3, respectively, were found to show *R* and *Q*^2^_test_ of 0.81 and 0.622. Although its *R*-value is slightly less, it showed the high predictive ability of the test compounds with a *Q*^2^_test_ of 0.622. Hence, Hypo #1 of Run No. 3 was considered to be the best model for further validation.

**Fig. 1:**
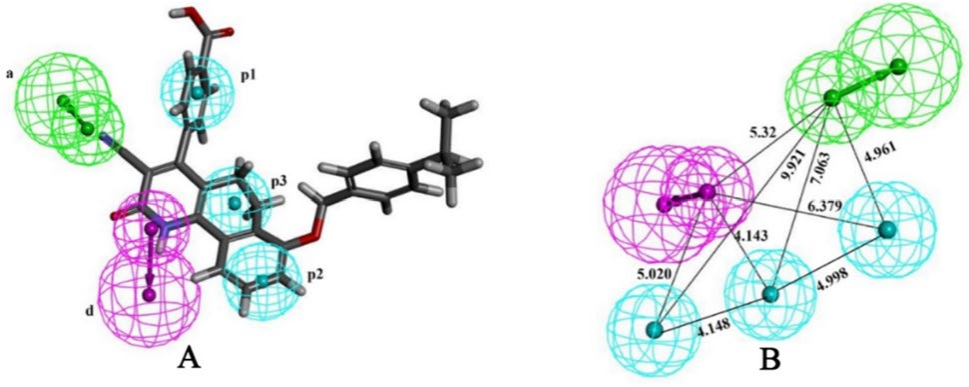
**A** Best pharmacophore model mapped with most active ligand, **B** Inter-feature distance between ‘a’, ‘d’, ‘p1’, ‘p2’ and ‘p3’

The 3D representation of the selected model is depicted in Fig. 1A, which shows the importance of one of each HB acceptor and donor and three hydrophobic regions in 3D space for MTK inhibition. The inter-feature distance was estimated to get an idea of the spatial arrangement of the revealed features, which is given in Fig. 1B. It can be seen that the pharmacophoric features ‘a’ and ‘p3’ were situated with the highest distance of 9.921, whereas the closest two features were found to be ‘d’ and ‘p2’ with a distance of 4.143. Hence, it can be postulated that the five pharmacophoric features revealed with their inter-feature distances might be crucial for the inhibitory activity of MTK.

### Validation of the selected model

It is necessary to validate the hypogen models to check their robustness, wide applications, and predictive ability. To explore the statistical robustness of the selected mode, it was validated using internal, cost-value analysis, test set, Fischer’s randomization test, and decoy set.

**Fig. 2:**
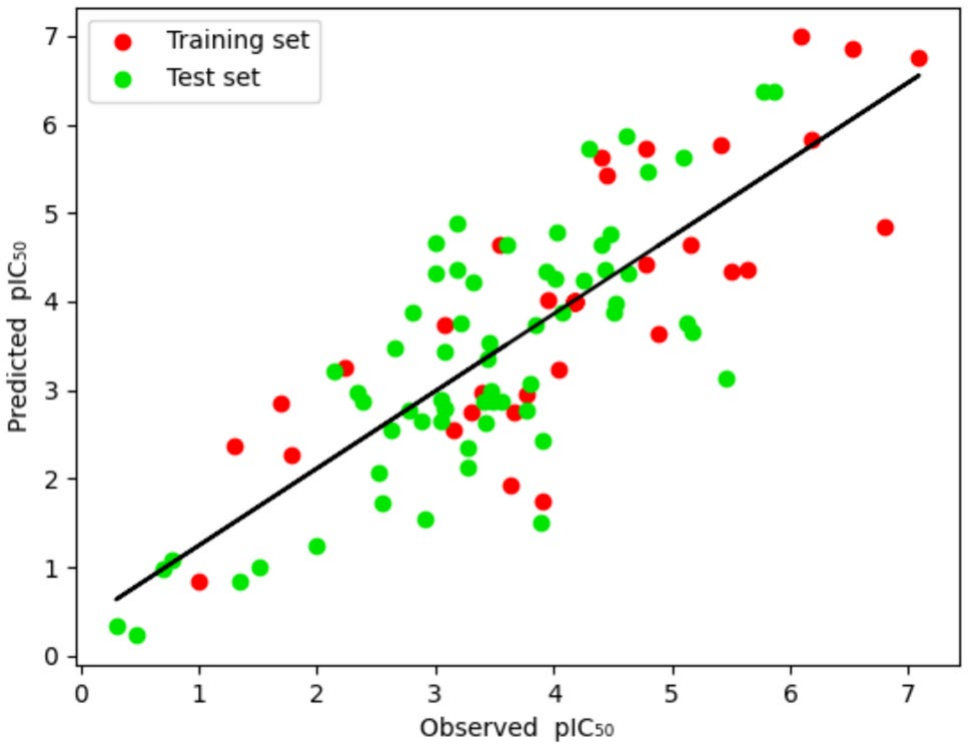
Observed and predicted inhibitory activity

To assess the credibility of the developed model, the pIC_50_ (log[IC_50_] × 10^−9^) of the training set molecule was estimated, which is given in Fig. 2. The best-fitting curve reflects the closeness and distinctness between the training set compounds’ observed and predicted biological activity values. Further, the cross-validated correlation coefficient (*Q*^2^_train_) was calculated from the observed and estimated biological activity of training set compounds, which was found to be 0.608 with the rmse of 1.250. The high *Q*^2^ undoubtedly explains that the selected model is robust and has excellent predictive ability for training set compounds.

The inhibitory activity of the test compounds was predicted using the selected pharmacophore model. The observed and predicted pIC_50_ of the test compounds are given in Fig. 2. The *R*^2^_pred_ for the test compounds was found to be 0.615, suggesting the closeness between the observed and predicted pIC50. Further, the cross-validated correlation coefficient (*Q*^2^_test_) along with the RMSE (Root Mean Square Error) of the test set molecules were calculated, and they were found to be 0.622 and 0.875, respectively. The above predictive assessment parameters exposed the statistical robustness of the pharmacophore model.

HypoGen provides several key parameters during the hypothesis generation phase in evaluating pharmacophore models, encompassing diverse cost functions and RMSD. The Δcost, total cost, null cost, fixed cost, and configuration cost are notable among these cost functions. The Δcost, determined to be 148, substantiates that the model’s development does not result from random chance. In the context of establishing a statistically robust model, it is imperative for the disparity between fixed and total costs to be minimized. In the present investigation, this disparity is quantified at 131. For a pharmacophore model to be deemed consistent and well-validated, the configuration cost ideally should be below 17. The configuration cost for Hypo #1 of Run no. 3 (as presented in Table 2) is recorded at 16.80. Further scrutiny involves the assessment of RMSD, a pivotal metric gauging the disparity between observed and predicted infectivity. In the case of Hypo #1 of Run no. 3, the RMSD is computed at 1.69. This comprehensive cost-value analysis unequivocally supports the credibility and reliability of the pharmacophore model under consideration.

**Table 2:** Binding energy of TM1, TM2, TM3, TM4, M31 and TMP and interacting amino acids of TMK

Compds	Binding energy (kcal/mol)			Binding interactions	
	ADV	PLANTS	K_Deep_	Hydrogen bonds	Other interactions
TM1	− 8.90	− 99.780	− 5.75	ARG95	TYR103, TYR165 (Hydrophobic bonds), Phe70 (Pi-Stacking)
TM2	− 9.20	− 113.970	− 5.27	ARG95, GLU166	PRO37, TYR39, ALA49, LEU52, TYR165 (Hydrophobic bonds), Phe70 (Pi-Stacking), GLU166 (Salt bridge)
TM3	− 9.80	− 116.927	− 6.10	PRO37, ARG95	ARG14, ALA35, PRO37, TYR39, ALA49, SER99, ASN100, TYR103 (Hydrophobic bonds), Phe70 (Pi-Stacking)
TM4	− 10.20	− 99.530	− 5.50	ARG95	ARG14, PRO37, ALA49, LEU52, PHE70, SER99, ASN100, TYR103, TYR165 (Hydrophobic bonds), Phe70 (Pi-Stacking)
M31	− 8.90	− 88.610	− 5.21	TYR39, GLN41	ALA35, PRO37, ALA49, PHE70, SER99, ASN100, TYR103, TYR165 (Hydrophobic bonds), Phe70 (Pi-Stacking)
TMP	− 8.60	− 91.520	− 5.06	TYR39, ARG95	PRO37, TYR39, ALA49, PHE70, SER99, ASN100, TYR103, TYR165 (Hydrophobic bonds), Phe70 (Pi-Stacking)

**Fig. 3:**
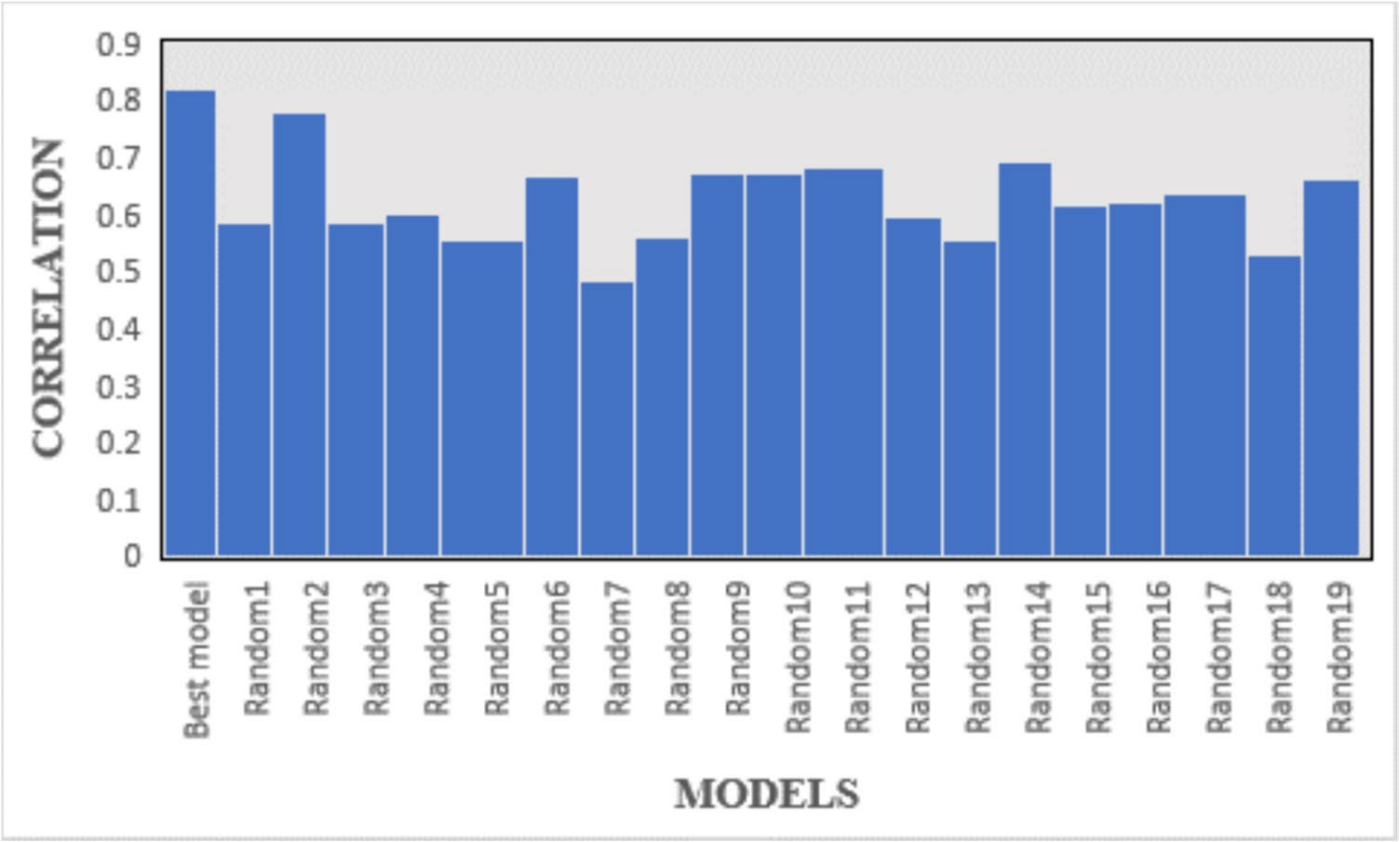
The total cost of 19 randomized runs and the best model in Fischer’s randomization test

**Fig. 4:**
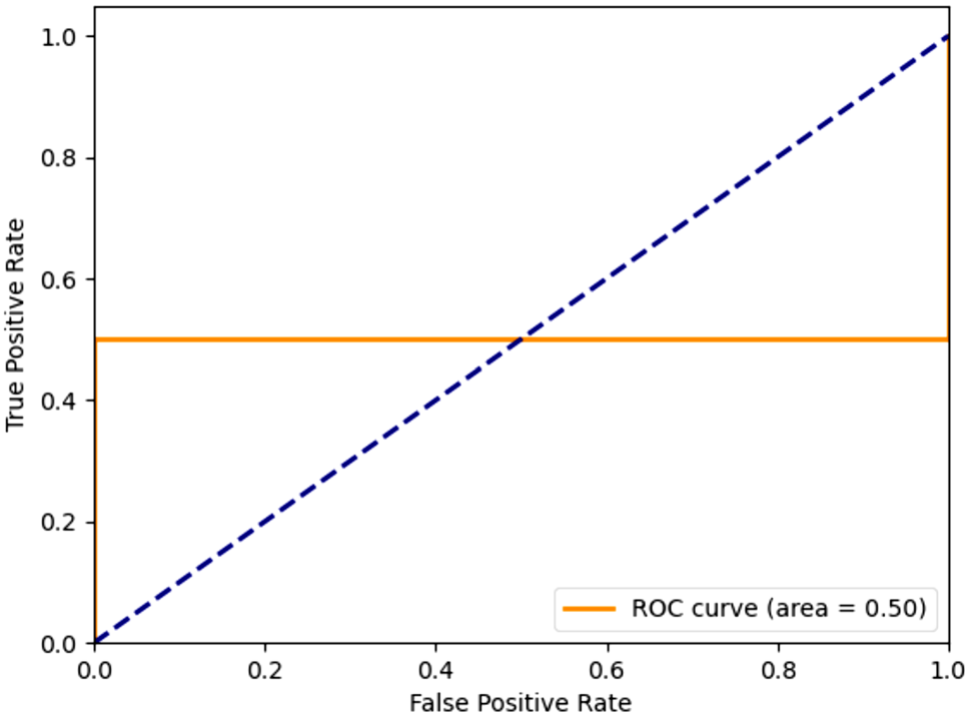
Decoy validation of 25 most active compounds along with 1250 decoys

The quality assessment of the chosen hypothesis was conducted through Fischer’s randomization test to establish a confidence level. Specifically, the selected hypothesis (Hypo #1 of Run no. 3) was subjected to a 95% confidence level, wherein the observed inhibitory activity of the training set compounds was randomized across 19 distinct spreadsheets, each generating a hypothesis. The highest correlation coefficient and total cost for each spreadsheet were compiled and presented in Fig. 4. Upon careful examination of Fig. 3, it becomes evident that none of the randomized iterations demonstrated predictive capabilities equivalent to or surpassing that of Hypo #1 of Run no. 3. The highest correlation coefficient observed among the 19 trials was 0.779, with an average value of 0.618. Notably, the highest and average correlation values from the 19 trials were markedly lower compared to Hypo #1 of Run no. 3. Moreover, the total costs across all 19 trials were consistently higher than that of Hypo #1 of Run no. 3. In summary, the analysis of correlation coefficients and total costs for both Hypo1 of Run no. 3 and the 19 randomized trials yield the conclusive inference that the selected hypothesis exhibits superior predictive capacity and was not fortuitously generated.

A collection of 1250 decoy compounds was obtained using the 25 highly active compounds out of the 94 active compounds of the BindingDB database using the DUD-E database to assess the screening efficacy of the chosen pharmacophore model. Subsequently, a composite dataset was created by combining 25 active compounds with the decoy compounds, and this composite set was subjected to screening using Hypo #1 of Run no. 3. The best-fit score of 1250 decoy compounds was generated using the Ligand-based pharmacophore mapping tool in DS. The screening outcomes revealed an accuracy of 0.550 and a precision of 0.560. ROC plot, illustrating the TP rate of active compounds versus the FP rate of decoys, was generated, which is given in Fig. 4. The calculated AUC was determined to be 0.500.

It can be seen that the selected model (Hypo #1 of Run no. 3) exhibited good predictive and statistically robust in nature. Hence, the aforementioned findings and explanations robustly affirm that the pharmacophoric features encapsulated and are adept at effectively screening and identifying active molecules within molecular databases.

#### Virtual screening

Virtual screening, a potent and efficient technique, is a valuable alternative to high-throughput screening methodologies for identifying potential molecules. In this pursuit, well-validated pharmacoinformatics models, including QSAR, pharmacophore, or molecular docking, are instrumental for lead identification within molecular databases. Utilizing Hypo #1 of Run no. 3, an exploration of the kinase library of the Enamine database, comprised 64,000 compounds, was undertaken to identify potential TMK inhibitors. The ‘Search Database’ functionality of DS was employed for screening, with ‘Search Method’ and ‘Limit Hits’ set to ‘Best’ and ‘Best N,’ respectively. Then, the sorted compounds were subjected to the ‘Ligand Pharmacophore Mapping’ protocol of DS, with a maximum omitted feature set to ‘0.’ After mapping 64000 compounds, a total of 2977 compounds were fetched.

### Molecular docking using Autodock vina

**Fig. 5:**
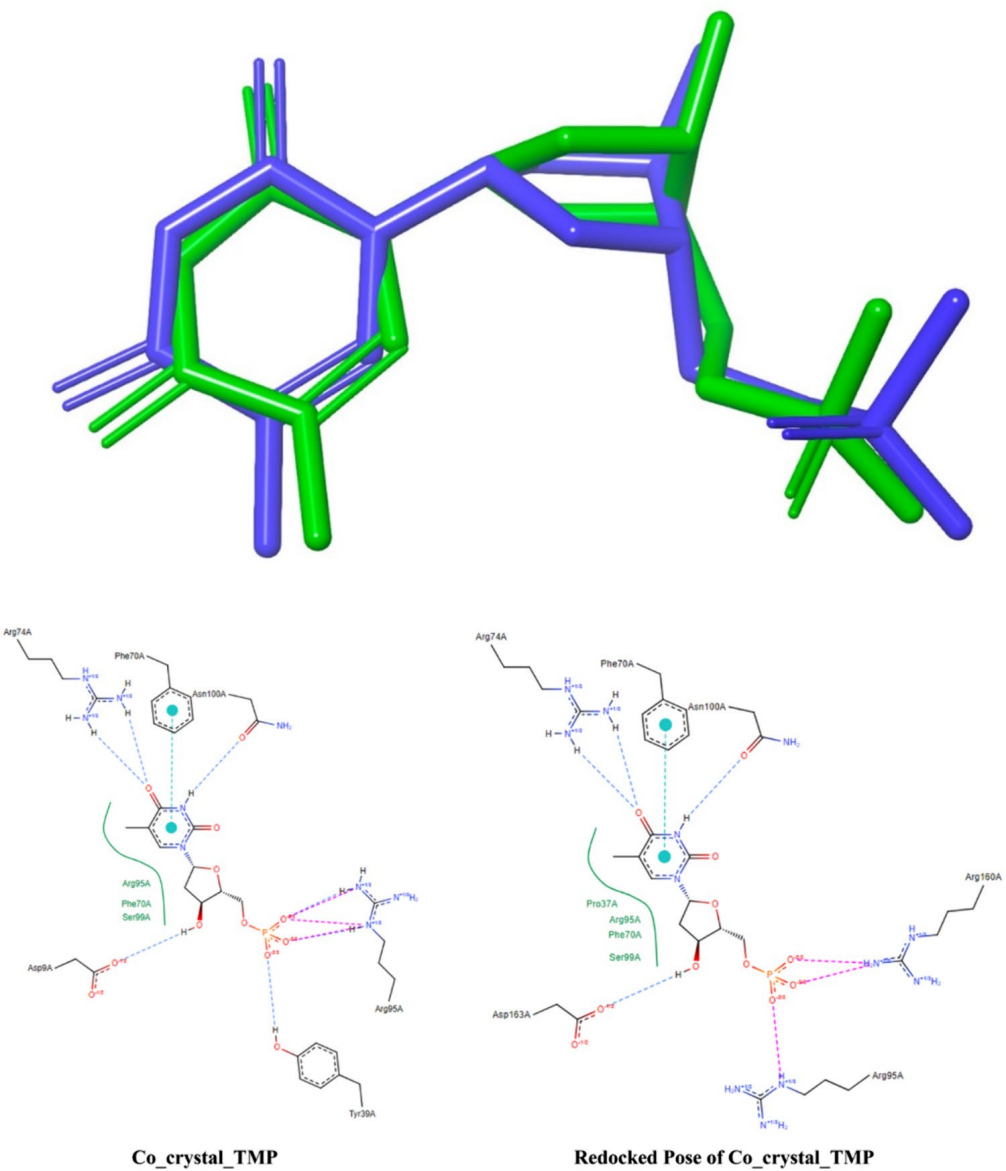
Superimposition of co-crystal (Green) conformer and best-docked (Blue) pose of TMP and 2D structures of co-crystal TMP along with the redocked pose of co-crystal TMP

Molecules found after the ligand pharmacophore mapping-based screening were considered for screening using the molecular docking study. Before docking the selected molecules, the molecular docking protocol was validated using the redocking approach. In particular, the 2D structure of TMP was redrawn and docked at the active site of TMK protein. The best pose from the docking study was extracted and superimposed on the co-crystal conformer. The binding energy of TMP was found to be − 8.60 kcal/mol. The RMSD after superimposition was found to be 1.300 Å, which clearly validated the considered molecular docking protocol. Hence, it can be assumed that the selected docking protocol can generate comparable conformation as crystallization. The superimposed docked and co-crystal TMP along with their 2D structure interactions are given in Fig. 5. Here the interacting amino acids are similar of the redocked pose and the co-crystal ligand with the protein complex of TMK including hydrogen bonds, hydrophobic interactions and cationic ions.

**Fig. 6:**
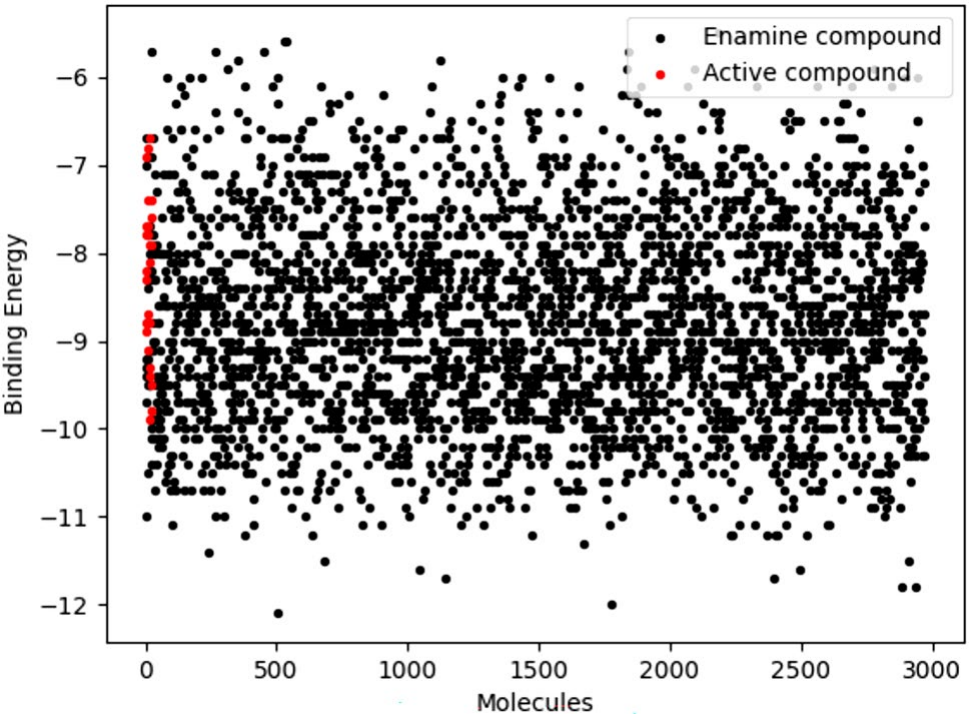
The binding energy of active molecules and Enamine molecules from ADV

The validated docking protocol was used to dock the molecules retained after pharmacophore-based screening. In addition, 25 active molecules obtained from the considered dataset were also docked. The docking process involved each compound being docked with the prepared TMK protein, and subsequent binding affinity calculations were conducted. The binding energy of the above two sets is given in Fig. 6.

The binding affinity of the most active molecule (M31) in the dataset was found to be − 8.90 kcal/mol, which is considered the threshold for narrowing down the chemical space. Using the above criteria, 2175 molecules were retained and considered for further analysis.

### ADME and toxicity parameters prediction using pkCSM

The molecules that remained after the screening through molecular docking study were considered for the pharmacokinetic and toxicity assessment using the pkCSM web server. The pharmacokinetic and toxicity parameters of each molecule were explored, and molecules having intestinal absorption ≥ 30, skin permeability ≤  − 2.5, BBB ≤  − 1, central nervous system (CNS) permeability ≤  − 3.0, AMES toxicity = No, hepatotoxicity = No, skin sensitivity = No, minnow toxicity ≥  − 0.3, and maximum tolerated dosage ≤ 0.477 were retained for the further analyses. After screening the molecules through the above criteria, it was seen that 217 molecules fulfilled the pharmacokinetic profile and showed non-toxic in nature.

#### Molecular docking using PLANTS and absolute binding free energy estimation using K_Deep_

**Fig. 7:**
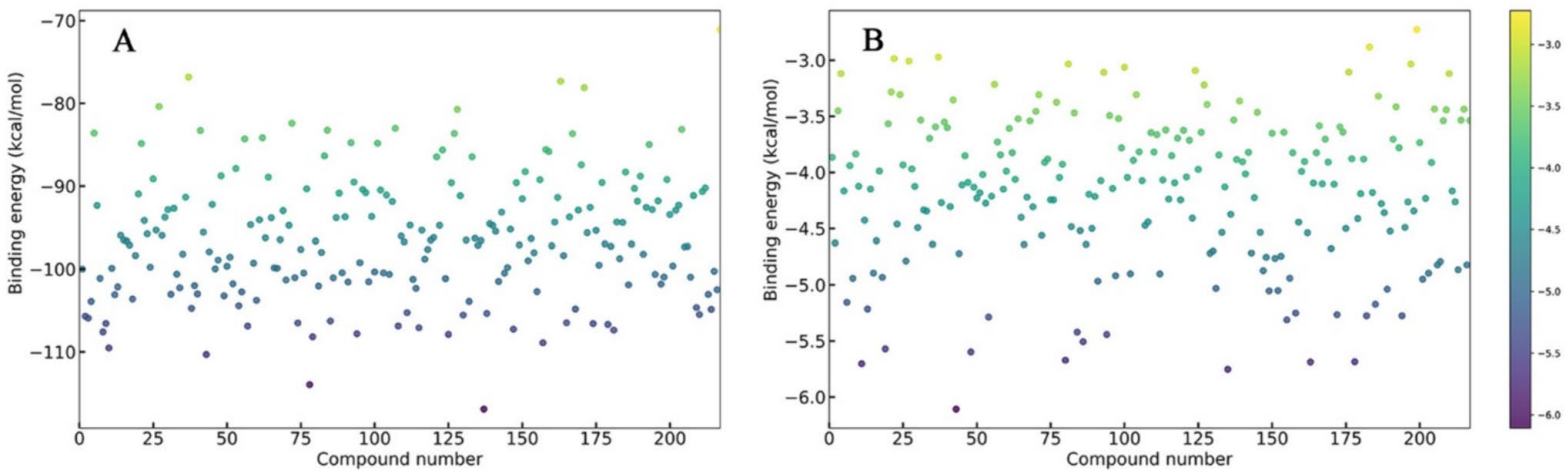
The binding energy of Enamine molecules against TMK. **A** PLANTS, **B** K_Deep_

The molecules found after the pharmacokinetic and toxicity analyses were used for molecular docking using PLANTS and the absolute binding energy estimation through K_Deep_ simultaneously. The binding energy and absolute binding energy of 217 molecules are given in Figs. 7A and B, respectively. It was found that each of the 217 molecules was shown a high negative binding energy and absolute binding energy that indicated the strong affinity of the molecules toward the TMK.

**Fig. 8:**
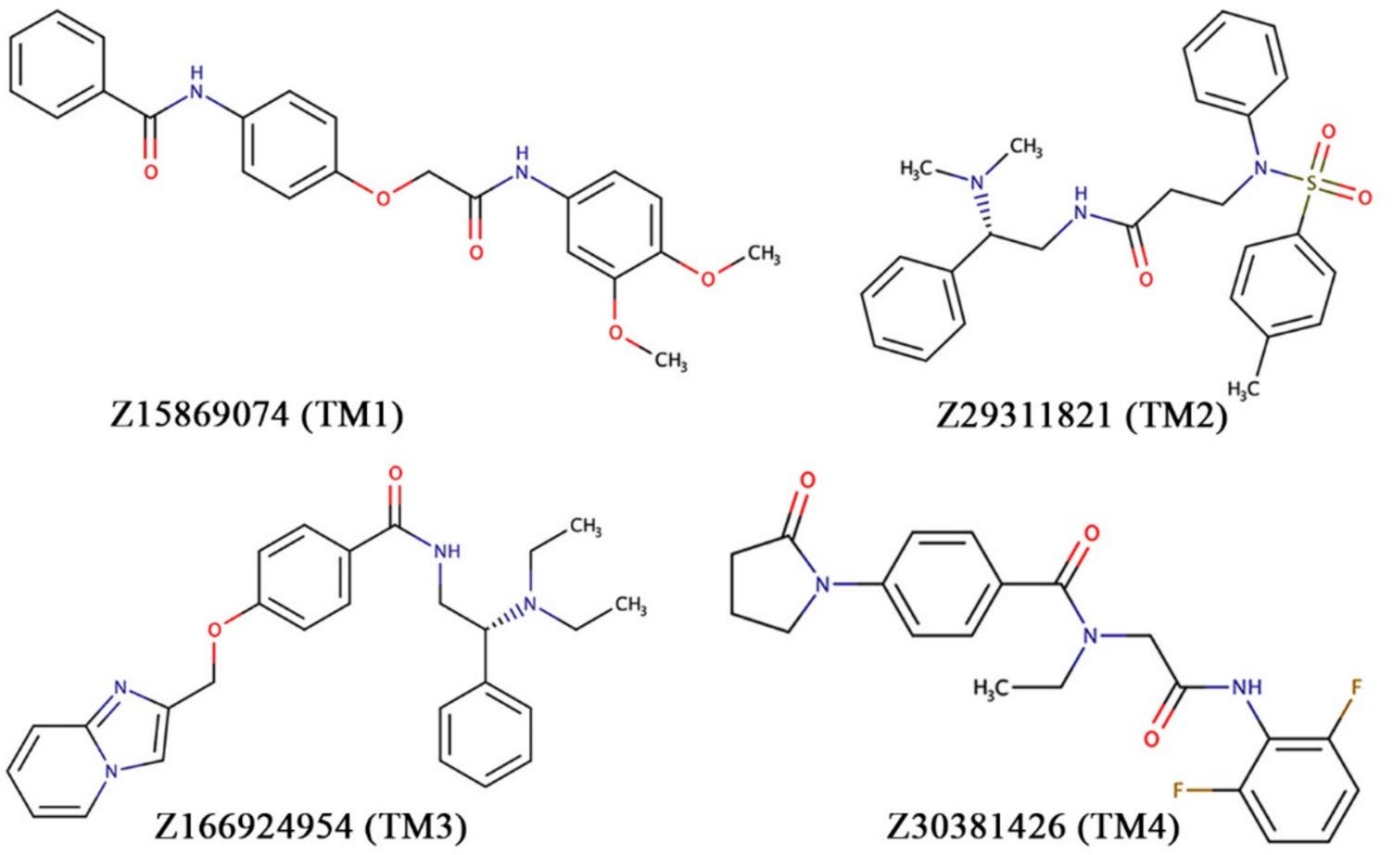
Two-dimensional representation of final TMK inhibitors

Moreover, the binding energy and absolute binding energy of M31 and TMP were also calculated, and it was found to be − 88.610 and − 5.21 kcal/mol, and − 91.520 and − 5.06 kcal/mol, respectively. Hence, to select the better affinity molecules, the binding energy (− 88.610 kcal/mol) and absolute binding energy (− 5.21 kcal/mol) of M31 were considered as thresholds. By applying the above threshold values, four molecules from the Enamine database showed better affinity towards TMK than the M31 and TMP. Hence, these four molecules (Z15869074, Z29311821, Z166924954, and Z30381426) were considered to be promising chemical entities for TMK inhibition, further assessed through the binding interactions and MD simulation studies. A two-dimensional (2D) representation of the final proposed molecules is given in Fig. 8. For simplicity, from now onwards, Z15869074, Z29311821, Z166924954, and Z30381426 will known as TM1, TM2, TM3, and TM4, respectively.

#### Binding interactions

**Fig. 9:**
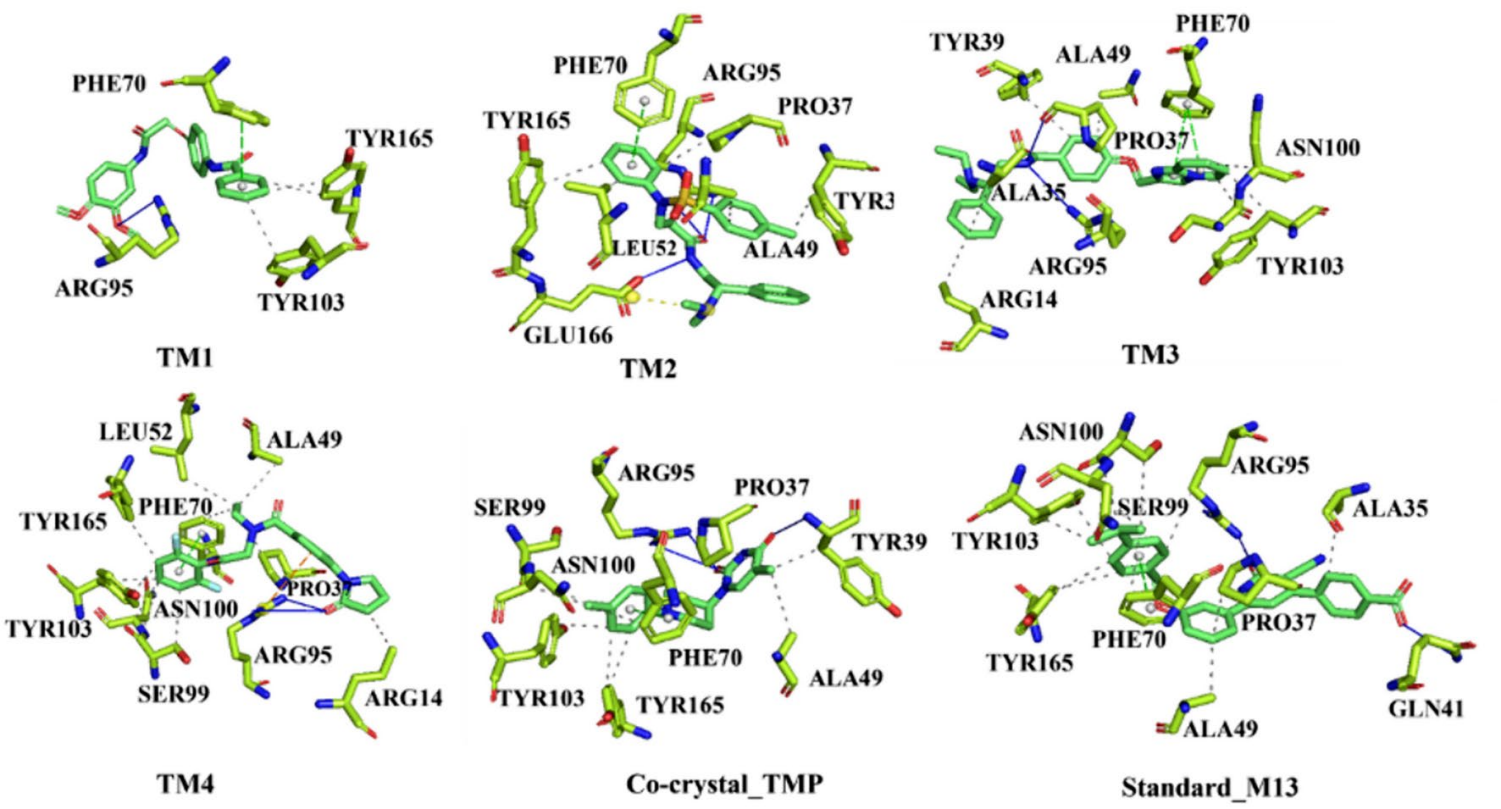
3D Binding interactions profile of TM1, TM2, TM3, TM4, M31 and TMP with TMK protein

The binding interactions of the proposed molecules, along with M1 and TMP, were explored through the PLIP web server, and the binding interaction profile is given in Fig. 9. Moreover, the binding interacting amino residues with corresponding binding interaction types are given in Table 2. It can be seen that ARG95 played an important role in the hydrogen bond interaction with all the proposed molecules and TMP. Only M31 formed a hydrophobic interaction with ARG95 instead of the hydrogen bond. TYR39 was found to form hydrogen bonds with M31 and TMP, but it failed to establish any hydrogen bond connection with the proposed molecules.

It can be noticed that the above amino acid (TYR39) was connected to TM2 and TM2 along with TMP through hydrophobic interactions. Hydrophobic amino acid Pro37 was seen to interact with TM3 through both hydrogen bonds and hydrophobic interactions, while the same was successfully connected with TM2, TM4, M31, and TMP through potential hydrophobic interactions. GLN41 was the lone amino acid that connected M31 via hydrogen bond interaction. Both hydrogen bond and hydrophobic connections were seen to form by the polar amino acid GLU166 with TM2. The non-polarity nature of ALA49 helped to establish hydrophobic contact with all the proposed molecules, M31 and TMP, except for TM1. It can be seen that TM2 and TM4 were found to have the potential to connect with LEU52 and TYR165 through hydrophobic contact. Further, it can be noticed that TYR165 formed hydrophobic interactions with TM1, M31, and TMP. Non-polar PHE70 was potentially connected with all four proposed molecules, M1 and TMP, through pi-stacking. Except for TM2, all molecules, including M31 and TMP, were seen to connect with TYR103 via hydrophobic contacts. Both polar amino residues, SER99 and ASN100, were found to be critical to connect with TM3, TM4, M31, and TMP. PHE70 was revealed as a crucial amino acid of TMK to form hydrophobic interaction with TM4, M31, and TMP. Both ARG14 and ALA35 were connected to TM3, while ARG14 and ALA35 were separate TM4 and M31, respectively. Apart from the above, ARG95 and GLU166 were connected with M31 and TM2 through hydrophobic and salt bridge, respectively. Overall, it can be seen that all four proposed molecules formed a significant number of crucial binding interactions in the form of hydrogen bonds and hydrophobic contacts, which suggest a strong association between the final molecules and TMK. Several interacting amino acids were found to be common between proposed molecules, and M31 and TMP portrayed the efficient binding of the molecules at the active site.

**Fig. 10:**
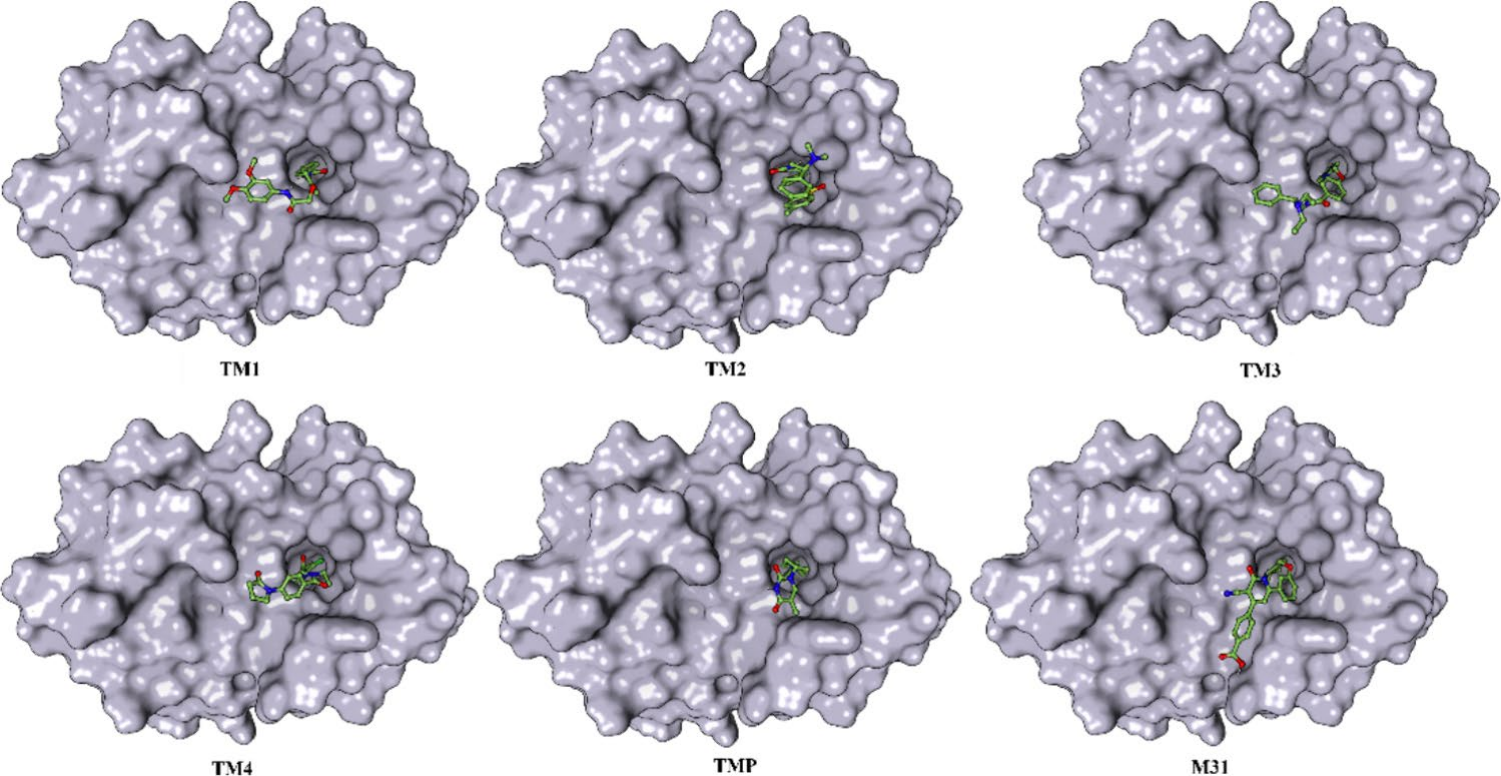
Surface view representation of best-docked pose of TM1, TM2, TM3, TM4, M31 and TMP in TMK protein

The binding energy from ADV, PLANTS, and K_Deep_ of TM1, TM2, TM3, TM4, M31, and TMP is given in Table 2. The higher binding affinity of proposed molecules compared to M31 and TMP exposed the potentiality of the molecules as TMK inhibitors. In order to check the binding mode of each proposed molecule along with M31 and TMP, the surface view representation of the bending binding pose is given in Fig. 10. On close inspection of the binding mode, it can be seen that all molecules perfectly fit and occupied the active site cavity of the TMK.

#### Molecular dynamics simulation

The dynamic behavior and stability of TMK protein bound with all the potential inhibitors, and the standard inhibitor M31 and co-crystal ligand TMP were evaluated using an all-atoms 100 ns MD simulation study. The best docking poses of each inhibitor attached to TMK protein were subjected to a conventional MD simulation study. The MD simulation trajectories were assessed to determine different trajectories, analyzing parameters such as protein backbone and ligand RMSD, RMSF, RoG, inter-molecular hydrogen bonds, FEL, and the binding free energy using the MM-GBSA technique. Values of each trajectory analyzing parameters in terms of minimum, maximum, and average values for RMSD of both the TMK backbone and ligand, RMSF, and RoG are given in Table 3.

**Table 3:** Statistical parameters obtained from MD simulation trajectories

Parameters		TM1	TM2	TM3	TM4	TMP	M31
Backbone RMSD (nm)	Average	0.207	0.205	0.216	0.170	0.186	0.233
	Maximum	0.343	0.348	0.390	0.226	0.274	0.331
	Minimum	0.000	0.000	0.000	0.000	0.000	0.000
Ligand RMSD (nm)	Average	0.158	0.200	0.244	0.189	0.178	0.208
	Maximum	0.257	0.334	0.359	0.280	0.253	0.269
	Minimum	0.000	0.000	0.000	0.000	0.000	0.000
RMSF (nm)	Average	0.142	0.143	0.116	0.124	0.142	0.132
	Maximum	0.466	0.596	0.416	0.371	0.533	0.504
	Minimum	0.057	0.053	0.047	0.050	0.058	0.050
RoG (nm)	Average	1.690	1.943	1.680	1.682	1.680	1.691
	Maximum	1.750	1.733	1.730	1.755	1.740	1.758
	Minimum	1.627	1.628	1.630	1.630	1.632	1.631

### Protein backbone RMSD analysis

**Fig. 11:**
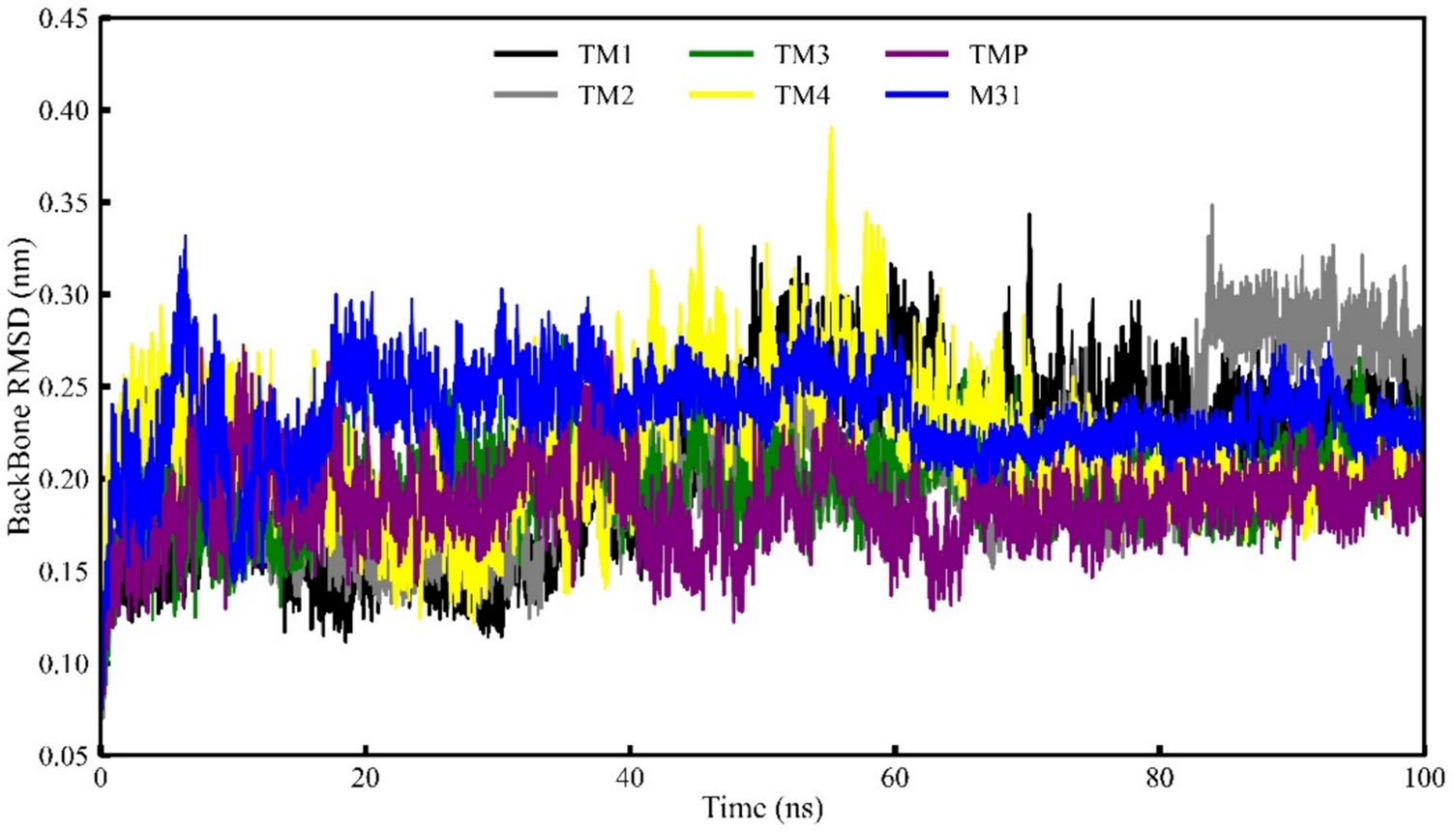
Protein backbone RMSD of four selected compounds, TMP and M31 with protein TMK

Protein backbone RMSD was utilized to evaluate the deviation of the Mtb TMK protein backbone when bound to various potential inhibitory compounds. Figure 11 illustrates the TMK protein backbone RMSD values for each frame generated during a 100 ns MD simulation involving four distinct inhibitory compounds. The analysis revealed that TMK protein backbone complexes with the proposed inhibitor compounds TM3 and TM2 exhibited consistent RMSD values with minimal variations, indicating robust stability throughout the simulation period. In the case of the inhibitory compound TM4, slight RMSD values oscillated until approximately 55 ns, but these fluctuations were not sustained throughout the entire simulation duration. A detailed examination disclosed that the higher RMSD value for TM3 did not signify conformational changes in the protein backbone structure. Notably, the increased RMSD gradually stabilized, reaching an equilibration state immediately after approximately 65 ns, and maintained a consistent level without further fluctuations in protein backbone RMSD values until the conclusion of the simulation run. The average TMK backbone RMSD was found to be 0.207, 0.205, 0.216, 0.170, 0.186 and 0.233 nm for TM1, TM2, TM3, TM4, TMP and M31, respectively. Hence, from low average backbone RMSD along with the consistent variation of the backbone during MD simulation expressed strong evidence of stability of the each system.

### Ligand RMSD analysis of Mtb TMK

**Fig. 12:**
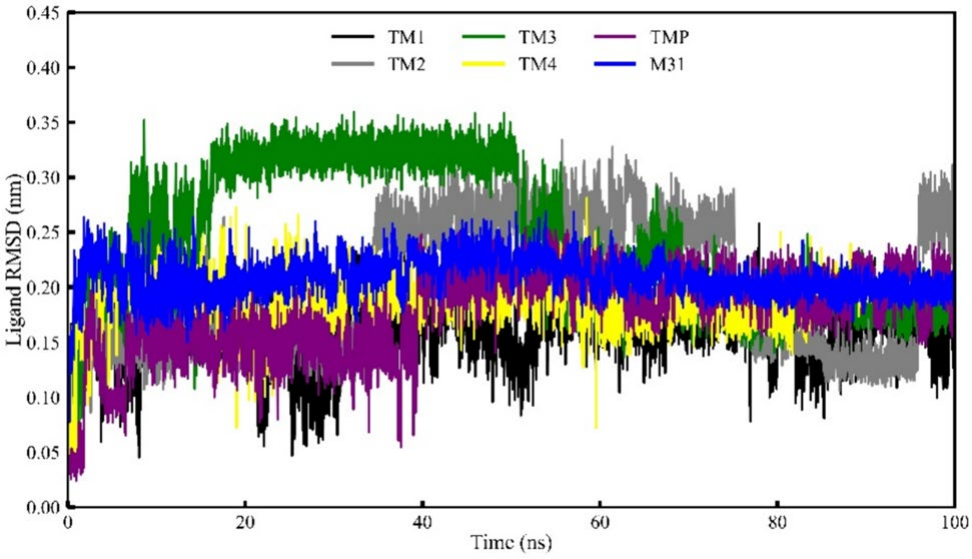
Ligand RMSD of four selected compounds, TMP and M31 bound with protein TMK

Ligand RMSD calculations were performed on the atoms of all compounds, revealing analogous RMSD profiles for two specific inhibitory compounds (TM1 and TM4), which remained consistently stable throughout the entire simulation period. In contrast, the compound TM4 exhibited a minor fluctuation during a specific time interval (Fig. 12). Specifically, the RMSD values for TM4 were slightly diverged from the beginning of the simulation run until approximately 20 ns, followed by being stable during short time intervals (~ 23–37 ns and ~ 63–100 ns). Compound TM3 reached the highest average RMSD value of 0.35 nm among all compounds over the entire simulation period. The visual representation indicates that all proposed inhibitory compounds maintained tight positional conformations within the active site cavity of the Mtb TMK protein until the conclusion of the simulation run, suggesting nearly stable conformations for the protein–ligand complexes. From Table 3, the range of the ligand RMSD variation can be seen as 0.257, 0.334, 0.359, 0.280, 0.253, and 0.269 nm for TM1, TM2, TM3, TM4, TMP, and M31, respectively. Overall, the consistently low RMSD values of the ligand RMSD indicated the molecular systems’ overall stability throughout the MD simulation.

### RMSF analyses of the Mtb TMK bound with inhibitory compounds

**Fig. 13:**
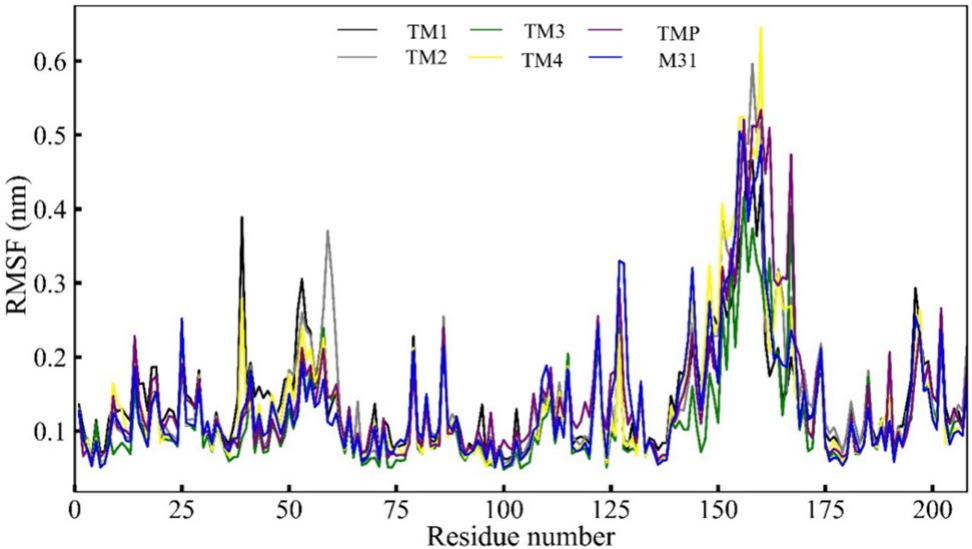
RMSF analyses of four selected compounds, TMP co-crystal and m1 with protein TMK

The RMSF value of individual residues of the Mtb TMK protein, plotted throughout simulations, is depicted in Fig. 13. Despite observing elevated RMSF values for all compounds interacting with the TMK protein, slight fluctuations on a large scale were noted. The RMSF plot illustrates that each compound bound to the Mtb TMK protein exhibited residue fluctuations within the 0.05–0.60 nm range. Table 3 provides the maximum, minimum, and average RMSF values derived from MD simulation trajectories for each compound associated with the Mtb TMK protein. Analysis revealed that amino acid residues spanning approximately from 14 to 26, 36 to 46, 25 to 75, and 125 to 175 exhibited higher-scale fluctuations when interacting with inhibitor compounds TM1 and TM4, compared to other compounds. These pronounced fluctuations are likely attributed to structural conformations of the protein, particularly in the loop regions extending from residues ~ 185–200 and 220–230, which are situated distantly from the binding pocket. The observed RMSF fluctuations in these specific amino acid residues were anticipated, as the studied compounds were not found to engage in molecular binding interactions at these protein regions (Table 4).

**Table 4:** MMGBSA analyses of the four selected compounds, M31 and co-crystal TMP

Compound	Binding free energy (kcal/mol)						
	Δ*E*_VDW_	Δ*E*_Ele_	Δ*G*_GB_	Δ*G*_Surf_	Δ*G*_Gas_	Δ*G*_Solv_	Δ*G*_Bind_
TM1	− 30.80 (± 4.98)	− 24.18 (± 4.98)	40.05 (± 10.94)	− 4.61 (± 0.58)	− 54.97 (± 12.94)	35.44 (± 10.65)	− 19.53 (± 4.40)
TM2	− 33.74 (± 4.13)	− 76.89 (± 20.84)	90.86 (± 17.95)	− 5.54 (± 0.52)	− 110.63 (± 20.20)	85.31 (± 17.77)	− 25.32 (± 5.19)
TM3	− 19.67 (± 4.49)	− 112.7 (± 31.15)	133.77 (± 1.81)	− 3.98 (± 0.53)	− 132.35 (± 31.10)	129.79 (± 28.62)	− 2.55 (± 5.03)
TM4	− 27.49 (± 5.79)	− 13.21 (± 12.34)	25.76 (± 12.08)	− 3.81 (± 0.82)	− 40.70 (± 15.35)	21.95 (± 11.55)	− 18.75 (± 5.72)
TMP	− 17.35 (± 8.01)	− 30.73 (± 22.65)	35.40 (± 20.13)	− 2.64 (± 1.27)	− 48.08 (± 27.7)	32.76 (± 18.99)	− 15.31 (± 9.53)
M31	− 20.21 (± 5.32)	− 29.04 (± 18.54)	42.41 (± 19.24)	− 4.21 (± 2.54)	− 62.14 (± 23.14)	86.21 (± 19.24)	− 11.05 (± 11.74)

### RoG analyses of the Mtb TMK

**Fig. 14:**
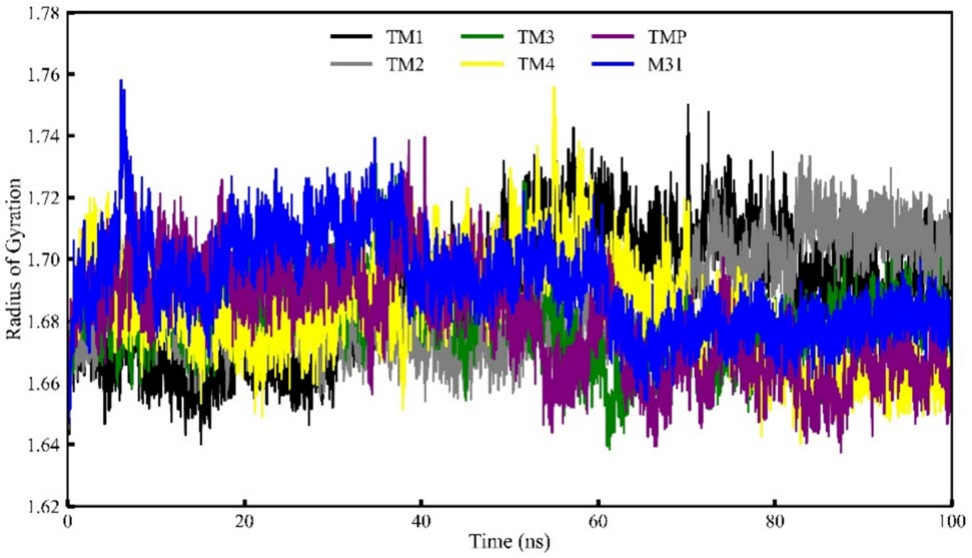
Radius of gyration of four selected compounds, TMP co-crystal and M31 with protein TMK

The RoG was employed as a crucial parameter in analyzing MD simulation trajectories to evaluate the structural compactness and folding organization of the Mtb TMK protein in its compound-bound state. The average RoG value for compounds TM1, TM2, TM3, TM4, M31, and TMP was determined as 1.690, 1.943, 1.680, 1.682, 1.691 and 1.680 nm, respectively. The RoG values for each frame of the Mtb TMK protein complexed with proposed inhibitor compounds were plotted against time, as illustrated in Fig. 14.

The RoG values indicated that the structural compactness of the Mtb TMK protein complex remained consistently folded throughout the simulation period for all food compounds, with no discernible changes in the RoG plot except for the standard compound (M31). Specifically, the Mtb TMK protein structure exhibited a relatively similar pattern of RoG values when bound to all proposed inhibitor compounds, displaying minimal fluctuations of very low magnitude across the entire MD simulation duration. The inhibitor compounds TM1 and TM4 showed slight fluctuations in RoG values, suggesting alterations in the protein folding state or reduced compactness. The limited variation in RoG values for the proposed inhibitor compounds implies that these compounds when bound to the Mtb TMK protein, maintained stable folding and structural compactness throughout the simulation period.

### H-bond interaction analyses of the Mtb TMK bounds with inhibitor compounds during MD simulation

**Fig. 15:**
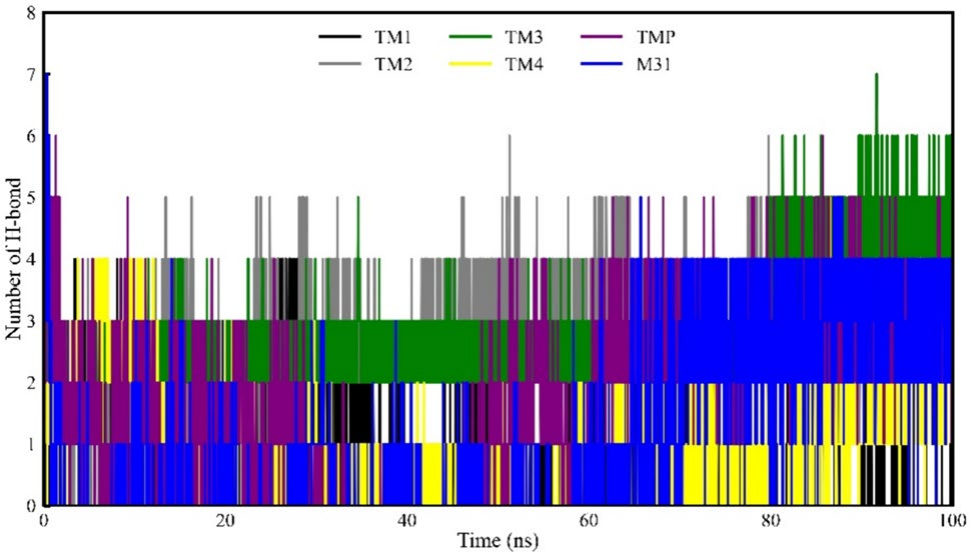
H-bond interactions of four selected compounds, TMP co-crystal and M31 with protein TMK

The hydrogen bond (H-bond) interaction analysis was conducted throughout the entire simulation period for all compounds, including the reference compound M31 and TMP; the results are presented in Fig. 15. Notably, inhibitor compounds TM3 and TM4 exhibited the highest occurrences of H-bond formations during the simulation, generating four H-bond interactions for a specific time duration. The other two inhibitory compounds, TM1 and TM2, also engaged in H-bond interactions during the MD simulation, albeit with relatively fewer instances, i.e., 2–3 and 0–1, respectively. Intriguingly, docking-based intermolecular interaction analyses in the present study corroborated these findings, revealing similar numbers of H-bond interactions with the proposed food compounds. In complex molecular structures, H-bonding is crucial in mediating interactions, influencing structural changes, and governing kinetic processes.

### MM-GBSA-based binding free energy estimation for protein–ligand complexes

The assessment of binding energy using the MM-GBSA approach, derived from the MD simulation trajectories, was considered to be a more precise and rigorous method. All proposed inhibitory molecules, including the standard compound, exhibited robust binding affinity towards the Mtb TMK protein. Specifically, the binding free energy of compounds TM1, TM2, TM3, TM4, TMP, and M31 was determined to be − 19.533, − 25.319, − 2.552, − 18.753, − 15.313 and − 11.05 kcal/mol, respectively. Notably, TM3 demonstrated a relatively lower binding free energy score of − 15.313 kcal/mol compared to the other proposed inhibitor compounds and TMP. Additionally, three of the proposed inhibitor compounds (TM1, TM2, and TM4) exhibited slightly similar or comparable binding free energies to the TMP. This similarity may be attributed to the presence of analogous chemical sub-structures in different proposed inhibitor compounds and similarities like their binding interactions with several amino acid residues of the Mtb TMK protein. Significant intermolecular interactions, including hydrogen bonds, hydrophobic interactions, electrostatics, and *π*-stacking interactions, were observed among various atoms of inhibitor compounds and distinct sub-site residues of the Mtb TMK protein. These interactions contribute to the comparable binding free energies observed for the identified compounds. In summary, the obtained binding free energy estimations from the simulated dynamic trajectories indicate that all proposed molecules exhibit substantial binding interaction affinity towards the Mtb TMK protein.

### FEL analyses for protein–ligand complexes

**Fig. 16:**
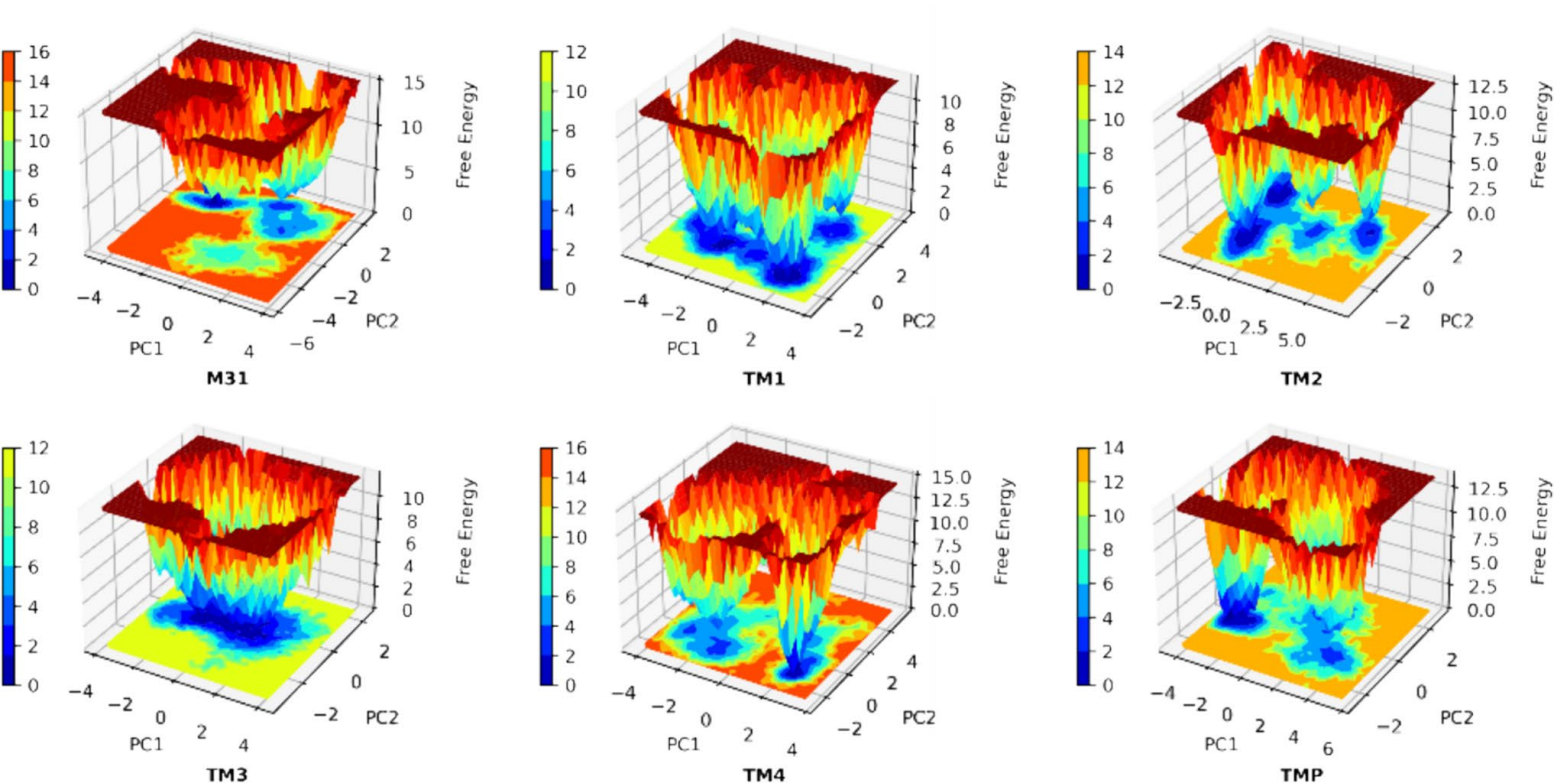
Free Energy Landscape analyses of the four selected compounds, m1 and TMP co-crystal

The study employs Free Energy Landscape (FEL) analysis, depicted in Fig. 16, to explore the dynamic behavior of the TMK protein upon binding with small molecules. The FEL plot reveals multiple local minima on the protein surface, corresponding to distinct conformational states and their respective free energies. Regions of low free energy, indicative of stable conformations, are observed as “energy basins” in the FEL plot. These basins signify thermodynamically favorable states where TMK adopts specific structural configurations during molecular dynamics simulations. Notably, all investigated compounds and M31 demonstrate pronounced energy basins, highlighting the consistent stability of TMK across various ligand interactions. These findings deepen our understanding of how TMK dynamically responds to ligand binding, offering insights crucial for drug discovery and protein engineering strategies.

## Conclusion

This study aimed to comprehensively understand the structural features governing the inhibitory activity against Mtb TMK. Through the analysis of a selected set of TMK inhibitors, a robust 3D QSAR pharmacophore model was constructed. This model elucidated essential features, comprising two hydrophobic sites, one negatively charged site, one acceptor site, and one hydrogen bond donor site, crucial for TMK inhibition and development of potential molecules. Further, the Enamine database was screened using the above pharmacophoric features. Our study underscores the promising utility of the developed pharmacophore model in predicting the inhibitory activity (pIC50) of test compounds against Mycobacterial Thymidylate kinase (TMK). With an R2pred value of 0.615, our model demonstrates a substantial ability to accurately estimate the observed pIC50 values, indicating that approximately 61.5% of the variability in experimental data can be explained by our predictions. This statistical robustness is further supported by a cross-validated correlation coefficient (Q2test) of 0.622 and a Root Mean Square Error (RMSE) of 0.875, affirming the reliability and consistency of our predictive framework. These findings are pivotal in accelerating the identification and optimization of potential TMK inhibitors, essential for combating drug-resistant strains of tuberculosis (TB). Moving forward, our validated pharmacophore model holds promise for guiding future drug discovery efforts, potentially offering new avenues to address the global health challenge posed by TB and its resistant forms. By leveraging molecular docking simulations alongside the analysis of pharmacokinetic and toxicity profiles of screened compounds, four promising Mtb TMK molecules were proposed. The potentiality of the molecules was exposed through high binding affinity through multiple docking algorithms. Several critical binding interactions between proposed molecules and the TMK showed a strong association between them. The acceptable pharmacokinetic profile and non-toxic nature of the final molecules revealed their acceptability to the human body. Several statistical parameters from the MD simulation trajectories explained the dynamic stability. High negative binding free energy from the MD simulation trajectories strongly favor their affinity towards MTK. Hence, the proposed molecules might be crucial for treating or managing TB.

## Supplementary Information

Below is the link to the electronic supplementary material.

Supplementary file1 (DOCX 45 KB)

## Data Availability

No datasets were generated or analysed during the current study.
