# Supplementary material for: Identification of mycobacterial Thymidylate kinase inhibitors: a comprehensive pharmacophore, machine learning, molecular docking, and molecular dynamics simulation studies
Source: Mol Divers. 2024 Aug 16;28(4):1947–64. doi: 10.1007/s11030-024-10967-w (PMC11449957; doi:10.1007/s11030-024-10967-w)
Supplement: Supplementary file 1 — Supplementary file1 (DOCX 45 KB) [file 11030_2024_10967_MOESM1_ESM.docx]

**Identification of Mycobacterial Thymidylate kinase inhibitors: A comprehensive pharmacophore, machine learning, molecular docking, and molecular dynamics simulation studies**

***Supplementary data***

Table S1. Training and test set molecules

| Mol no | SMILES | Set #1 | | Set #2 | | Set #3 | | Set #4 | |
| --- | --- | --- | --- | --- | --- | --- | --- | --- | --- |
|  |  | Tr | Ts | Tr | Ts | Tr | Ts | Tr | Ts |
| M1 | Cc1cn([C@H]2C[C@H](O)[C@@H](COP(=O)(O)OP(=O)(O)OP(=O)(O)OP(=O)(O)OP(=O)(O)OC[C@H]3O[C@@H](n4cnc5c(N)ncnc54)[C@H](O)[C@@H]3O)O2)c(=O)[nH]c1=O | Y | N | Y | N | Y | N | Y | N |
| M2 | CSc1cc(-c2ccccc2)nc(O)c1C#N | N | Y | Y | N | N | Y | Y | N |
| M3 | Cc1cn([C@H]2C[C@H](O)[C@@H](CO[P@@](=O)(O)O[P@@](=O)(O)O[P@@](=O)(O)O[P@@](=O)(O)OC[C@H]3O[C@@H](n4cnc5c(N)ncnc54)[C@H](O)[C@@H]3O)O2)c(=O)[nH]c1=O | N | Y | N | Y | N | Y | N | Y |
| M4 | Cn1c(=O)[nH]c2ncc(-c3cccc(C(=O)NCCCc4ccc(F)cc4)c3)cc21 | N | Y | Y | N | N | Y | N | Y |
| M5 | Cn1c(=O)[nH]c2ncc(-c3cccc(C(=O)NCCCc4ccc(C(F)(F)F)cc4)c3)cc21 | Y | N | N | Y | Y | N | N | Y |
| M6 | Cc1cn([C@H]2CCCN([C@H](C)c3ccc(C(=O)O)c(Oc4cccc(Cl)c4)c3)C2)c(=O)[nH]c1=O | N | Y | Y | N | N | Y | N | Y |
| M7 | CCC[C@H](c1ccc(C(=O)O)c(Oc2cccc(Cl)c2)c1)N1CCC[C@H](n2cc(C)c(=O)[nH]c2=O)C1 | Y | N | Y | N | N | Y | N | Y |
| M8 | Cc1cn([C@H]2CCCN([C@H](CC(C)(C)C)c3ccc(C(=O)O)c(Oc4cccc(Br)c4)c3)C2)c(=O)[nH]c1=O | N | Y | Y | N | N | Y | N | Y |
| M9 | CCC(C)(C)C[C@H](c1ccc(C(=O)O)c(Oc2cccc(Cl)c2)c1)N1CCC[C@H](n2cc(C)c(=O)[nH]c2=O)C1 | Y | N | N | Y | N | Y | N | Y |
| M10 | CCCCC[C@H](c1ccc(C(=O)O)c(Oc2cccc(Br)c2)c1)N1CCC[C@H](n2cc(C)c(=O)[nH]c2=O)C1 | N | Y | Y | N | N | Y | N | Y |
| M11 | CSc1cc(-c2cccs2)[nH]c(=O)c1C#N | N | Y | N | Y | Y | N | N | Y |
| M12 | CSc1cc(-c2cccc(Cl)c2)[nH]c(=O)c1C#N | N | Y | N | Y | N | Y | N | Y |
| M13 | COc1cccc(-c2cc(SC)c(C#N)c(=O)[nH]2)c1 | N | Y | Y | N | N | Y | N | Y |
| M14 | COc1ccc(-c2cc(SC)c(C#N)c(=O)[nH]2)cc1 | N | Y | N | Y | N | Y | Y | N |
| M15 | CSc1cc(-c2ccccn2)[nH]c(=O)c1C#N | N | Y | Y | N | N | Y | N | Y |
| M16 | COc1cccc(-c2cc(-c3ccc(C(=O)O)cc3)c(C#N)c(=O)[nH]2)c1 | N | Y | N | Y | Y | N | N | Y |
| M17 | COc1cccc(-c2cc(-c3cccc(C(=O)O)c3)c(C#N)c(=O)[nH]2)c1 | N | Y | Y | N | N | Y | Y | N |
| M18 | COc1cccc(-c2cc(-c3ccc(N)cc3)c(C#N)c(=O)[nH]2)c1 | N | Y | N | Y | N | Y | N | Y |
| M19 | COc1cccc(-c2cc(-c3cccc(N)c3)c(C#N)c(=O)[nH]2)c1 | N | Y | Y | N | N | Y | N | Y |
| M20 | CCNC(=O)Nc1ccc(-c2cc(-c3cccc(OC)c3)[nH]c(=O)c2C#N)cc1 | Y | N | Y | N | N | Y | N | Y |
| M21 | COc1cccc(-c2cc(-c3cccnc3)c(C#N)c(=O)[nH]2)c1 | N | Y | Y | N | N | Y | N | Y |
| M22 | COc1cccc(-c2cc(-c3ccc(NC(C)=O)cc3)c(C#N)c(=O)[nH]2)c1 | Y | N | Y | N | N | Y | N | Y |
| M23 | COc1cccc(-c2nc(SC)c(C#N)c(=O)[nH]2)c1 | N | Y | N | Y | Y | N | N | Y |
| M24 | CSc1cc(-c2cccc(Cl)c2)[nH]c(=O)c1C(N)=O | Y | N | N | Y | N | Y | Y | N |
| M25 | COc1cccc(-c2cc(-c3ccc(C(=O)O)cc3)c(C)c(=O)[nH]2)c1 | N | Y | N | Y | N | Y | Y | N |
| M26 | CC(C)c1ccc(COc2cccc(-c3cc(-c4ccc(C(=O)O)cc4)c(C#N)c(=O)[nH]3)c2)cc1 | N | Y | N | Y | Y | N | N | Y |
| M27 | N#Cc1c(-c2ccc(C(=O)O)cc2)c2c([nH]c1=O)-c1ccccc1CC2 | Y | N | N | Y | N | Y | N | Y |
| M28 | N#Cc1c(-c2ccc(C(=O)O)cc2)c2c([nH]c1=O)-c1cccc(O)c1CC2 | N | Y | Y | N | N | Y | N | Y |
| M29 | COc1cccc2c1CCc1c-2[nH]c(=O)c(C#N)c1-c1ccc(C(=O)O)cc1 | N | Y | N | Y | Y | N | N | Y |
| M30 | N#Cc1c(-c2ccc(C(=O)O)cc2)c2c([nH]c1=O)-c1cccc(-c3cn[nH]c3)c1CC2 | Y | N | N | Y | N | Y | Y | N |
| M31 | CC(C)c1ccc(COc2cccc3c2CCc2c-3[nH]c(=O)c(C#N)c2-c2ccc(C(=O)O)cc2)cc1 | Y | N | N | Y | N | Y | N | Y |
| M32 | CCNC(=O)Nc1ccc(-c2c3c([nH]c(=O)c2C#N)-c2cccc(OC)c2CC3)cc1 | Y | N | Y | N | N | Y | Y | N |
| M33 | N#Cc1c(-c2ccc(C(=O)NS(=O)(=O)c3ccccc3)cc2)c2c([nH]c1=O)-c1ccccc1OC2 | N | Y | N | Y | Y | N | N | Y |
| M34 | CS(=O)(=O)NC(=O)c1ccc(-c2c3c([nH]c(=O)c2C#N)-c2ccccc2OC3)cc1 | Y | N | N | Y | N | Y | Y | N |
| M35 | CCCc1cc2[nH]c(=O)ccc2c(C)n1 | N | Y | N | Y | N | Y | Y | N |
| M36 | CCc1cc2[nH]c(=O)c(C)c(C)c2c(CC)n1 | N | Y | N | Y | Y | N | N | Y |
| M37 | CCc1cc2[nH]c(=O)c(C#N)c(-c3ccccc3)c2c(C)n1 | Y | N | N | Y | N | Y | N | Y |
| M38 | CCCc1cc2[nH]c(=O)c(C#N)c(-c3ccccc3)c2c(C)n1 | N | Y | Y | N | N | Y | N | Y |
| M39 | CCc1cc2[nH]c(=O)c(C#N)c(-c3ccc(NS(C)(=O)=O)cc3)c2c(C)n1 | Y | N | N | Y | Y | N | N | Y |
| M40 | CCCc1cc2[nH]c(=O)c(C#N)c(-c3ccc(NS(C)(=O)=O)cc3)c2c(C)n1 | N | Y | N | Y | N | Y | Y | N |
| M41 | C[S+]([O-])c1cc(-c2cccs2)[nH]c(=O)c1C#N | N | Y | N | Y | Y | N | N | Y |
| M42 | C[S+]([O-])c1cc(-c2cccc(OC(F)(F)F)c2)[nH]c(=O)c1C#N | N | Y | Y | N | N | Y | N | Y |
| M43 | C[S+]([O-])c1cc(-c2cccc(Br)c2)[nH]c(=O)c1C#N | N | Y | Y | N | N | Y | N | Y |
| M44 | CS(=O)(=O)c1cc(-c2cccc(Br)c2)[nH]c(=O)c1C#N | N | Y | N | Y | Y | N | N | Y |
| M45 | CC(C)CCOc1cccc(-c2cc([S+](C)[O-])c(C#N)c(=O)[nH]2)c1 | N | Y | N | Y | N | Y | Y | N |
| M46 | CS(=O)(=O)c1cc(-c2cccc(OCc3cccc(Cl)c3)c2)[nH]c(=O)c1C#N | N | Y | N | Y | Y | N | N | Y |
| M47 | CC(C)c1ccc(COc2cccc(-c3cc([S+](C)[O-])c(C#N)c(=O)[nH]3)c2)cc1 | N | Y | N | Y | N | Y | N | Y |
| M48 | CS(=O)(=O)c1c2c([nH]c(=O)c1C#N)-c1ccccc1OC2 | N | Y | Y | N | N | Y | N | Y |
| M49 | Cc1nccc2nc(O)c(C#N)cc12 | Y | N | N | Y | N | Y | N | Y |
| M50 | O=C(O)c1cc(SSc2ccc([N+](=O)[O-])c(C(=O)O)c2)ccc1[N+](=O)[O-] | N | Y | N | Y | Y | N | N | Y |
| M51 | Cc1cn(C2CCN(Cc3cccc(Oc4ccccc4)c3)CC2)c(=O)[nH]c1=O | Y | Y | N | Y | N | Y | Y | N |
| M52 | CC(C)(C)OC(=O)N1CCN(C(=O)Cn2sc3ccccc3c2=O)CC1 | N | Y | N | Y | N | Y | Y | N |
| M53 | CCOC(=O)N1CCN(C(=O)Cn2sc3nc(C)cc(C)c3c2=O)CC1 | N | Y | N | Y | Y | N | N | Y |
| M54 | CC(C)(C)OC(=O)N1CCN(C(=O)Cn2sc3ncccc3c2=O)CC1 | Y | N | N | Y | N | Y | Y | N |
| M55 | Cc1cn(C2CCN(Cc3cccc(Oc4cccc(F)c4)c3)CC2)c(=O)[nH]c1=O | N | Y | Y | N | N | Y | N | Y |
| M56 | Cc1cn(C2CCN(Cc3ccnc(Oc4ccccc4)c3)CC2)c(=O)[nH]c1=O | N | Y | N | Y | Y | N | N | Y |
| M57 | Cc1cn(C2CCN(Cc3cccc(Oc4cccc(C(F)(F)F)c4)c3)CC2)c(=O)[nH]c1=O | N | Y | N | Y | N | Y | Y | N |
| M58 | COc1cccc(Oc2cccc(CN3CCC(n4cc(C)c(=O)[nH]c4=O)CC3)c2)c1 | N | Y | Y | N | Y | N | Y | N |
| M59 | Cc1cn(C2CCN(Cc3cccc(OC4CCCC4)c3)CC2)c(=O)[nH]c1=O | Y | N | Y | N | Y | N | Y | N |
| M60 | COc1ccc(Oc2cccc(CN3CCC(n4cc(C)c(=O)[nH]c4=O)CC3)c2)cc1 | N | Y | N | Y | Y | N | N | Y |
| M61 | Cc1cn(C2CCN(Cc3cccc(Oc4cccnc4)c3)CC2)c(=O)[nH]c1=O | N | Y | Y | N | N | Y | N | Y |
| M62 | Cc1cn(C2CCN(Cc3cccc(Oc4ccc(Cl)c(C(F)(F)F)c4)c3)CC2)c(=O)[nH]c1=O | Y | N | N | Y | N | Y | N | Y |
| M63 | Cc1cn(C2CCN(Cc3cccc(Oc4ccc(Cl)cc4)c3)CC2)c(=O)[nH]c1=O | N | Y | N | Y | Y | N | N | Y |
| M64 | Cc1cn(C2CCN(Cc3cccc(Oc4cccc(Cl)c4)c3)CC2)c(=O)[nH]c1=O | Y | N | N | Y | N | Y | N | Y |
| M65 | Cc1cn(C2CCN(Cc3cccc(Oc4cc(F)cc(F)c4)c3)CC2)c(=O)[nH]c1=O | Y | N | Y | N | N | Y | N | Y |
| M66 | Cc1cn(C2CCN(Cc3cccc(Oc4ccncc4)c3)CC2)c(=O)[nH]c1=O | N | Y | N | Y | N | Y | Y | N |
| M67 | Cc1cn(C2CCN(Cc3cc(Oc4ccccc4)ccn3)CC2)c(=O)[nH]c1=O | N | Y | N | Y | Y | N | N | Y |
| M68 | Cc1cn(C2CCN(Cc3cccc(Oc4cc(C(F)(F)F)cc(C(F)(F)F)c4)c3)CC2)c(=O)[nH]c1=O | Y | Y | N | Y | N | Y | Y | N |
| M69 | Cc1cn(C2CCN(Cc3cccc(Oc4cccc(Cl)c4)n3)CC2)c(=O)[nH]c1=O | N | Y | N | Y | N | Y | N | Y |
| M70 | Cc1cn(C2CCN(Cc3cccc(Oc4ccc(Cl)c(Cl)c4)c3)CC2)c(=O)[nH]c1=O | N | Y | Y | N | N | Y | N | Y |
| M71 | Cc1ccc(Oc2cccc(CN3CCC(n4cc(C)c(=O)[nH]c4=O)CC3)c2)cc1 | N | Y | N | Y | N | Y | Y | N |
| M72 | Cc1cn(C2CCN(Cc3cccc(Oc4ccc(F)c(F)c4)c3)CC2)c(=O)[nH]c1=O | N | Y | N | Y | N | Y | Y | N |
| M73 | Cc1cn(C2CCN(Cc3cccc(OC4CCCCC4)c3)CC2)c(=O)[nH]c1=O | N | Y | N | Y | N | Y | N | Y |
| M74 | Cc1cn(C2CCN(Cc3cncc(Oc4cccc(OC(F)(F)F)c4)c3)CC2)c(=O)[nH]c1=O | Y | N | N | Y | N | Y | Y | N |
| M75 | Cc1cn(C2CCN(Cc3cc(Oc4cccc(Cl)c4)ccn3)CC2)c(=O)[nH]c1=O | N | Y | N | Y | Y | N | N | Y |
| M76 | Cc1cn(C2CCN(Cc3cccc(Oc4cccc(OC(F)(F)F)c4)c3)CC2)c(=O)[nH]c1=O | N | Y | N | Y | N | Y | Y | N |
| M77 | Cc1cn(C2CCN(Cc3cncc(Oc4cc(Cl)cc(Cl)c4)c3)CC2)c(=O)[nH]c1=O | N | Y | N | Y | N | Y | Y | N |
| M78 | Cc1cn(C2CCN(Cc3cccc(Oc4ccccc4)n3)CC2)c(=O)[nH]c1=O | Y | N | N | Y | N | Y | N | Y |
| M79 | Cc1cn(C2CCN(Cc3cccc(Oc4ccc(O)cc4)n3)CC2)c(=O)[nH]c1=O | N | Y | N | Y | N | Y | N | Y |
| M80 | Cc1cn(C2CCN(Cc3ccnc(Oc4cccc(Cl)c4)c3)CC2)c(=O)[nH]c1=O | N | Y | N | Y | N | Y | N | Y |
| M81 | Cc1cn(C2CCN(Cc3cccc(Oc4ccccc4C(=O)O)n3)CC2)c(=O)[nH]c1=O | N | Y | N | Y | N | Y | N | Y |
| M82 | Cc1cn(C2CCN(Cc3cncc(Oc4ccccc4)c3)CC2)c(=O)[nH]c1=O | N | Y | N | Y | Y | N | N | Y |
| M83 | Cc1cn(C2CCN(Cc3cccc(Oc4cccc(O)c4)c3)CC2)c(=O)[nH]c1=O | N | Y | N | Y | Y | N | N | Y |
| M84 | Cc1cn(C2CCN(Cc3cncc(Oc4cccc(Cl)c4)c3)CC2)c(=O)[nH]c1=O | N | Y | N | Y | N | Y | N | Y |
| M85 | Cc1cccc(Oc2cccc(CN3CCC(n4cc(C)c(=O)[nH]c4=O)CC3)c2)c1 | Y | N | Y | N | N | Y | N | Y |
| M86 | Cc1cn(C2CCN(Cc3cc(Oc4cc(Cl)cc(Cl)c4)ccn3)CC2)c(=O)[nH]c1=O | Y | N | N | Y | Y | N | N | Y |
| M87 | Cc1cn(C2CCN(Cc3cccc(Oc4cc(Cl)cc(Cl)c4)c3)CC2)c(=O)[nH]c1=O | Y | N | Y | N | N | Y | N | Y |
| M88 | Cc1cn(C2CCN(Cc3cccc(Oc4ccccn4)c3)CC2)c(=O)[nH]c1=O | N | Y | N | Y | N | Y | Y | N |
| M89 | Cc1cn(C2C[C@H](O)[C@@H](COP(=O)(O)OP(=O)(O)OP(=O)(O)OP(=O)(O)OP(=O)(O)OP(=O)(O)OC[C@H]3OC(n4cnc5c(N)ncnc54)[C@H](O)[C@@H]3O)O2)c(=O)[nH]c1=O | Y | N | N | Y | Y | N | N | Y |
| M90 | Cc1cn(C2C[C@H](O)[C@@H](COP(=O)(O)CP(=O)(O)OP(=O)(O)OC[C@H]3OC(n4cnc5c(N)ncnc54)[C@H](O)[C@@H]3O)O2)c(=O)[nH]c1=O | N | Y | N | Y | Y | N | N | Y |
| M91 | Cc1cn(C2C[C@H](O)[C@@H](COP(=O)(O)OP(=O)(O)OP(=O)(O)OC[C@H]3OC(n4cnc5c(N)ncnc54)[C@H](O)[C@@H]3O)O2)c(=O)[nH]c1=O | Y | N | N | Y | N | Y | Y | N |
| M92 | Cc1cn(C2C[C@H](O)[C@@H](COP(=O)(O)CP(=O)(O)OP(=O)(O)OP(=O)(O)OP(=O)(O)OC[C@H]3OC(n4cnc5c(N)ncnc54)[C@H](O)[C@@H]3O)O2)c(=O)[nH]c1=O | N | Y | N | Y | N | Y | Y | N |
| M93 | Cc1cn(C2C[C@H](O)[C@@H](COP(=O)(O)CP(=O)(O)OP(=O)(O)OP(=O)(O)OP(=O)(O)OP(=O)(O)OC[C@H]3OC(n4cnc5c(N)ncnc54)[C@H](O)[C@@H]3O)O2)c(=O)[nH]c1=O | N | Y | Y | N | Y | N | Y | N |
| M94 | Cc1cn(C2C[C@H](O)[C@@H](COP(=O)(O)CP(=O)(O)OP(=O)(O)OP(=O)(O)OC[C@H]3OC(n4cnc5c(N)ncnc54)[C@H](O)[C@@H]3O)O2)c(=O)[nH]c1=O | Y | N | Y | N | Y | N | Y | N |

Table S2. Statistical data of pharmacophore developed from set #2-4

| **Run No.** | **Spacing** | **Unc.** | **Wt. Var.** | **R** | **Rmsd** | **Costs** | | | | |  |  |  |  |
| --- | --- | --- | --- | --- | --- | --- | --- | --- | --- | --- | --- | --- | --- | --- |
|  |  |  |  |  |  | **Total** | **Null** | **Fixed** | **∆** | **Config.** | **Features** | **Q^2^** |  |  |
| **Set #2** | | | | | | | | | | | | |  |  |
| 1 | 3 | 3 | 0.3 | 0.91 | 1.37 | 148 | 278 | 119 | 130 | 17.19 | 2xa, 3xp | 0.16 |  |  |
| 2 | 2.5 | 3 | 0.3 | 0.92 | 1.33 | 149 | 278 | 118 | 129 | 16.6 | a,2xp,r | 0.313 |  |  |
| 3 | 2 | 3 | 0.3 | 0.9 | 1.44 | 155 | 278 | 118 | 123 | 16.805 | a,2xp,r | 0.248 |  |  |
| 4 | 1.5 | 3 | 0.3 | 0.88 | 1.58 | 160 | 278 | 180 | 118 | 16.813 | d,3xp,r | 0.001 |  |  |
| 5 | 1 | 3 | 0.3 | 0.89 | 1.52 | 156 | 278 | 118 | 122 | 16.818 | d,3xp,r | 0.034 |  |  |
| 6 | 2.5 | 2.5 | 0.3 | 0.92 | 1.33 | 149 | 278 | 119 | 129 | 16.6 | a,p,2xr | 0.313 |  |  |
| 7 | 2.5 | 2 | 0.3 | 0.9 | 2.31 | 190 | 532 | 105 | 342 | 16.83 | a,p,2xr | 0.232 |  |  |
| 8 | 2.5 | 1.5 | 0.3 | 0.92 | 3.5 | 294 | 1372 | 88 | 1078 | 16.83 | d,3xp,r | 0.006 |  |  |
| **Set #3** | | | | | | | | | | | | |  |  |
| 1 | 3 | 3 | 0.3 | 0.76 | 2.33 | 208 | 295 | 119 | 87 | 17.37 | a,2xp,r | 0.3 |  |  |
|  |  |  |  |  |  |  |  |  |  |  |  |  |  | |
| 2 | 2.5 | 3 | 0.3 | 0.81 | 2.1 | 195 | 295 | 119 | 100 | 17.58 | a,p,2xr | 0.184 |  |  |
|  |  |  |  |  |  |  |  |  |  |  |  |  |  | |
| 3 f | 2 | 3 | 0.3 | 0.76 | 2.31 | 201 | 295 | 119 | 94 | 17.78 | a,d,3xp | 0.2 |  |  |
|  |  |  |  |  |  |  |  |  |  |  |  |  |  | |
| 4 | 1.5 | 3 | 0.3 | 0.81 | 2.09 | 189 | 295 | 119 | 106 | 17.79 | 2xa,3xp | 0.263 |  |  |
| 5 | 1 | 3 | 0.3 | 0.82 | 2.04 | 188 | 295 | 119 | 107 | 17.8 | a,d,3xp | 0.213 |  |  |
|  |  |  |  |  |  |  |  |  |  |  |  |  |  | |
| 6 | 3 | 2.5 | 0.3 | 0.76 | 2.33 | 208 | 295 | 119 | 87 | 17.37 | a,2xp,r | 0.296 |  |  |
|  |  |  |  |  |  |  |  |  |  |  |  |  |  | |
| 7 | 3 | 2 | 0.3 | 0.75 | 2.84 | 236 | 375 | 113 | 139 | 17.37 | 2xa,3xp | 0.268 |  |  |
| 8 | 3 | 1.5 | 0.3 | 0.85 | 2.97 | 249 | 577 | 105 | 328 | 17.37 | 2xa,3xp | 0.265 |  |  |
| 9 | 3 | 3 | 0.3 | 0.78 | 6.06 | 665 | 1503 | 89 | 838 | 17.37 | d,2xp,r | 0.236 |  |  |
|  |  |  |  |  |  |  |  |  |  |  |  |  |  | |
| **Set #4** | | | | | | | | | | | | |  |  |
| 1 | 3 | 1.5 | 0.3 | 0.76 | 5.04 | 545 | 1166 | 88.7 | 621 | 16.58 | d,3xp,r | 0.27 |  |  |
|  |  |  |  |  |  |  |  |  |  |  |  |  |  | |
| 2 | 2.5 | 1.5 | 0.3 | 0.77 | 5.35 | 525 | 1166 | 88 | 641 | 16.78 | a,3xp,r | 0.168 |  |  |
|  |  |  |  |  |  |  |  |  |  |  |  |  |  | |
| 3 f | 2 | 1.5 | 0.3 | 0.77 | 5.37 | 526 | 1166 | 89 | 640 | 16.97 | a,d,3p | 0.322 |  |  |
|  |  |  |  |  |  |  |  |  |  |  |  |  |  | |
| 4 | 1.5 | 1.5 | 0.3 | 0.79 | 5.15 | 492 | 1166 | 89 | 674 | 16.98 | d,p,2xr | 0.311 |  |  |
|  |  |  |  |  |  |  |  |  |  |  |  |  |  | |
| 5 | 1 | 1.5 | 0.3 | 0.77 | 5.44 | 535 | 1166 | 89 | 631 | 16.99 | a,3xp,r | 0.266 |  |  |
|  |  |  |  |  |  |  |  |  |  |  |  |  |  | |
| 6 | 2 | 1.5 | 0.3 | 0.77 | 5.37 | 526 | 1166 | 89 | 640 | 16.97 | a,d,3xp | 0.322 |  |  |
|  |  |  |  |  |  |  |  |  |  |  |  |  |  | |
| 7 | 2 | 2 | 0.3 | 0.78 | 3.07 | 251 | 461 | 105 | 210 | 16.97 | a,d,3xp | 0.421 |  |  |
| 8 | 2 | 2.5 | 0.3 | 0.8 | 2.26 | 191 | 309 | 113 | 118 | 16.97 | a,3xp,r | 0.287 |  |  |
|  |  |  |  |  |  |  |  |  |  |  |  |  |  | |
| 9 | 2 | 3 | 0.3 | 0.81 | 1.82 | 170 | 250 | 119 | 35 | 17.72 | 2xa,3xp | 0.336 |  |  |
|  |  |  |  |  |  |  |  |  |  |  |  |  |  |  |
